# A Short‐Read Amplicon Sequencing Protocol and Bioinformatic Pipeline for Ecological Surveillance of Dipteran Disease Vectors

**DOI:** 10.1111/1755-0998.14088

**Published:** 2025-02-18

**Authors:** Raquel Lima‐Cordón, Jason Travis Mohabir, Mohini Sooklall, Aina Martinez Zurita, Meg Shieh, Cheyenne Knox, Sabrina Gobran, Zachary Johnson, Margaret Laws, Ruchit Panchal, Reza Niles‐Robin, Horace Cox, Maria Eugenia Grillet, Jorge E. Moreno, Socrates Herrera, Martha Quinones, Angela M. Early, Jacob A. Tennessen, Daniel E. Neafsey

**Affiliations:** ^1^ Department of Immunology and Infectious Diseases Harvard T.H. Chan School of Public Health Boston Massachusetts USA; ^2^ Broad Institute Cambridge Massachusetts USA; ^3^ Vector Control Services Ministry of Public Health Georgetown Guyana; ^4^ Caribbean Public Health Agency Port of Spain Trinidad and Tobago; ^5^ Laboratorio de Biología de Vectores y Parásitos Instituto de Zoología y Ecología Tropical, Facultad de Ciencias, Universidad Central de Venezuela Caracas Venezuela; ^6^ Centro de Investigaciones de Campo Dr. Francesco Vitanza Instituto de Alto Estudios Dr., Arnoldo Gabaldon Tumeremo Bolivar Venezuela; ^7^ Centro de Investigación Científica Caucaseco Cali Colombia; ^8^ Departamento de Salud Pública Facultad de Medicina, Universidad Nacional de Colombia Bogotá Colombia

**Keywords:** amplicon sequencing, blood and plant meal, entomological surveillance, insecticide resistance, *plasmodium*, taxonomic identification, vector species

## Abstract

Vector control remains an important strategy worldwide to prevent human infection with pathogens transmitted by arthropods. Vector control strategies rely on accurate identification of vector taxa along with vector‐specific biological indicators such as feeding ecology, infection prevalence and insecticide resistance. Multiple ‘DNA barcoding’ protocols have been published over the past several decades to support these applications, generally relying on informal manual approaches such as BLAST to assign taxonomic identity to the resulting sequences. We present a standardised informatic pipeline for analysis of DNA barcoding data from dipteran vectors, VecTreeID, that uses short‐read amplicon sequencing (AmpSeq) coupled with sequence similarity assessment (BLAST) and an evolutionary placement algorithm (EPA‐ng) to achieve vector taxonomic identification, capture bionomic features (blood and plant meal sources), determine *Plasmodium* infection status (for anopheline mosquitoes) and detect target‐site insecticide resistance mutations. The VecTreeID pipeline provides uncertainty in assignment through identifications at varying levels of taxonomic rank, a feature missing from many approaches to DNA barcoding, but important given gaps and labelling problems in public sequence databases. We validated an Illumina‐based implementation of VecTreeID on laboratory and field samples, and find that the blood meal amplicons can detect vertebrate DNA sequences up to 36 h post‐feeding, and that short‐read sequencing data are capable of sensitively detecting minor sequences in DNA mixtures representing multi‐species blood or nectar meals. This high‐throughput VecTreeID approach empowers researchers and public health professionals to survey and control arthropod disease vectors consistently and effectively.

## 1 Introduction

Infectious diseases spread by hematophagous arthropod vectors have significant impacts worldwide through induced mortality, socioeconomic burden and impaired individual performance (Bloom and Cadarette 2019; Sach and Malaney 2002). These vector‐borne diseases include dengue, yellow fever, West Nile, chikungunya, plague, Chagas, leishmaniasis and malaria, among others (Tolle 2009; Gubler 2010) and result in millions of deaths annually (World Health Organization 2014a; World Health Organisation., 2014b; Wilson et al. 2020). The most effective way to prevent vector‐borne diseases is through vector‐control strategies that target the species most important for disease transmission. Such strategies are informed by surveillance programs that gather vital information about disease dynamics through systematic sampling of arthropod vectors. Some of the most important entomological surveillance measures include occurrence and density, feeding profiles, biting sites, parasite/pathogen infection status and insecticide susceptibility (World Health Organization 2018). Thus, the effectiveness of surveillance programs requires not only accurate identification of vectors to determine their epidemiological importance, but also assessment of their biology to facilitate strategic control actions.

For dipteran vectors, morphological species identification is useful and cost‐effective but it requires extensive training and becomes challenging or impossible when dealing with damaged specimens, imperfect preservation and cryptic species complexes (Wilke et al. 2016; Erlank et al. 2018; Farlow et al. 2020). To validate or replace morphological identifications, DNA‐based ‘barcoding’ methods are now standard (Hill and Crampton 1994; Walton et al. 1999). Unlike morphology, molecular information not only provides insights into species diversity but also provides details about vector bionomics, infection status and insecticide resistance while being less sensitive to storage conditions (Farlow et al. 2020). DNA‐based methods primarily utilise the Polymerase Chain Reaction (PCR) to generate amplicons of standardised short loci, which are either evaluated via electrophoresis (Santolamazza et al. 2008; Jeyaprakasam et al. 2022) or sequenced as barcodes for species classification (Farlow et al. 2020; Batovska et al. 2016). The resulting sequences are then compared against a reference sequence library, and the identity of specimens is determined based on their close similarity to reference sequences (Hebert et al. 2003; Hajibabaei et al. 2007).

Molecular barcoding of insect vectors has transitioned from Sanger dideoxy‐based sequencing to multiplexed Illumina sequencing, through which PCR amplicons from several loci across many samples can be genotyped simultaneously, and the need to subclone PCR products to resolve heterozygosity is unnecessary. A recent series of studies has presented protocols for Illumina barcoding of insect specimens, either for individual loci (Batovska et al. 2018) or as amplicon panels (Rodgers et al. 2017; Reeves et al. 2018; Abbasi et al. 2018; Batovska et al. 2018; Cannon et al. 2021; Collins et al. 2022; Makunin et al. 2022; Boddé et al. 2022; Campos et al. 2022; Acford‐Palmer, Andrade, et al. 2023; Acford‐Palmer, Phelan, et al. 2023; Acford‐Palmer, Campos, et al. 2023). These new amplicon panels are invaluable for vector biology and highlight the effectiveness and potential of this approach for resolving species complexes, molecular insecticide screening, tracking parasite infection and revealing vector ecology. However, their scope of use tends to be highly focused, targeting key vector loci in a single species or species complex (Campos et al. 2022; Collins et al. 2022; Acford‐Palmer, Andrade, et al. 2023; Acford‐Palmer, Phelan, et al. 2023; Acford‐Palmer, Campos, et al. 2023), taxonomic distinctions within a narrow clade transmitting a particular infectious agent (e.g., anophelines of the *Cellia* and *Anopheles* subgenera and *Plasmodium* parasites (Makunin et al. 2022), vector identification in the context of a specific pathogen (Batovska et al. 2018)), or non‐insect DNA detection without simultaneous insect DNA assessment (Rodgers et al. 2017; Reeves et al. 2018; Abbasi et al. 2018; Cannon et al. 2021). Thus, these panels are most useful for researchers who have already narrowed the vector to a relatively small taxonomic window and/or have a focused question, who have high‐quality DNA that can be expected to amplify reliably, and who possess the bioinformatic skills to interpret the results. Practical application in the field will often require a more versatile, comprehensive and user‐friendly tool. Ideally, a vector surveyor in any part of the world should be able to take an unknown insect and obtain actionable information about species identification, adaptive polymorphisms, infection status, and ecological interaction with food sources, without special bioinformatics training. Furthermore, the limits of interpretation should be clearly defined, preventing misinterpretation of negative or ambiguous results due to poor DNA quality, cryptic taxa, or database insufficiency.

Here, we present an Illumina‐based amplicon sequencing (AmpSeq) protocol and bioinformatic analysis pipeline, VecTreeID, for assessing dipteran vector identifications and bionomics simultaneously. Our approach features two major advantages over previous vector AmpSeq panels: (1) breadth of amplicon targets assessing vector bionomics, and (2) replicable and objective interpretation of results due to a bioinformatic pipeline and thoroughly calibrated sensitivity and performance data. The loci we validated for this panel and associated bioinformatic workflows enable users to: (1) identify vector species present and their geographic distribution, (2) gain insights into the vertebrate and plant meal sources of various dipteran taxa, shedding light on vector ecology and behaviour, (3) detect and identify *Plasmodium* infections, (4) screen for mutations associated with target–site insecticide resistance and (5) utilise this data‐driven research approach to inform local vector control strategies and surveillance.

## 2 Materials and Methods

### 2.1 Amplicon Panel Development

**FIGURE 1:**
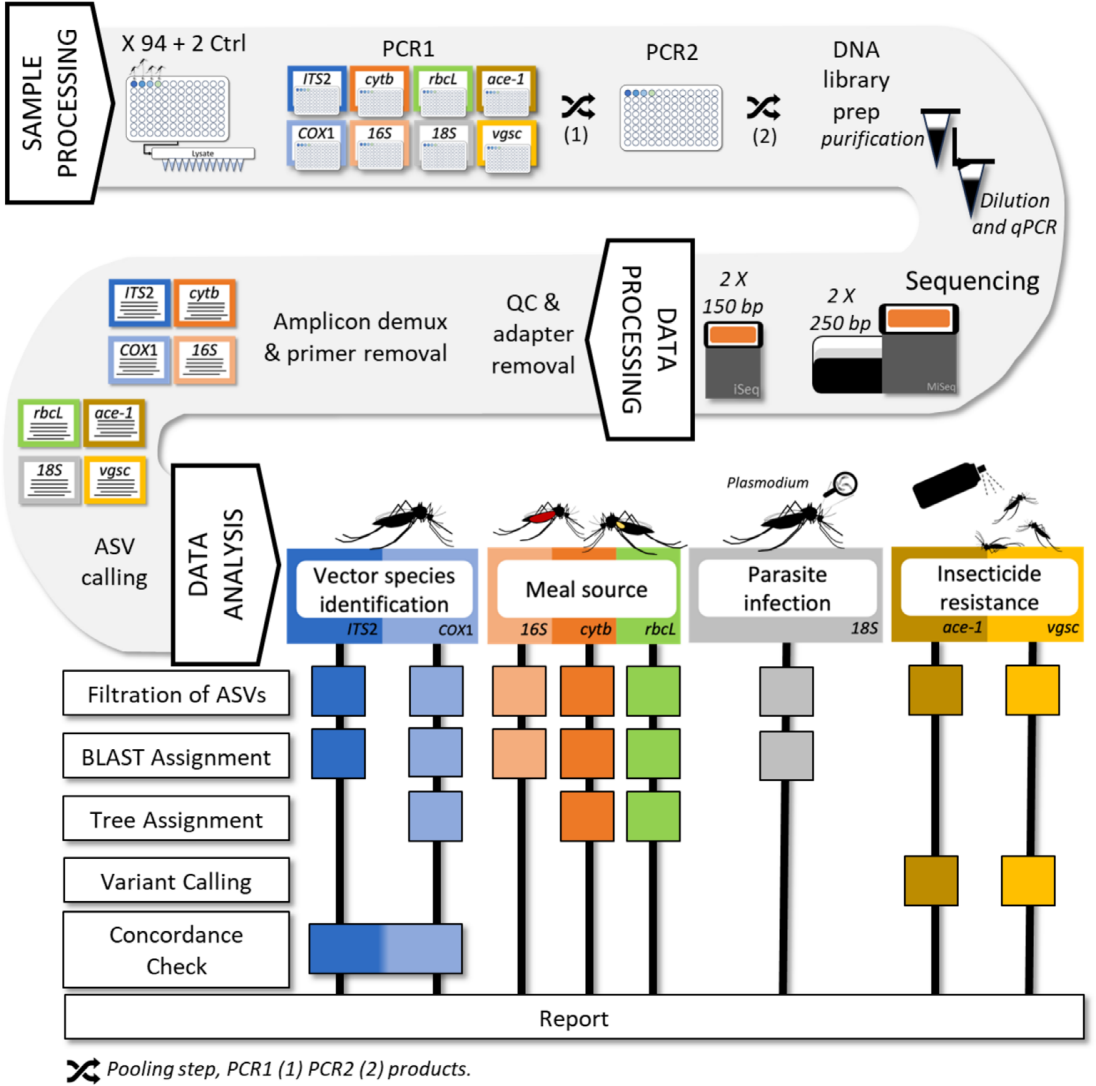
Amplicon sequencing and data processing workflow. Vector samples undergo lysis, PCR1 and PCR2 with Illumina dual indexing. Sequencing data are processed using DADA2 for quality control, demultiplexing, merging and calling of amplicon sequence variants (ASVs). Data analysis performs taxonomic assignment for the vector and any detected plant or blood meals and provides presence/absence information for *Plasmodium* infection and insecticide resistance markers.

We assessed the performance of eight amplicons initially designed for Sanger dideoxy sequencing or other AmpSeq panels in an Illumina‐based amplicon sequencing protocol (Figure 1; Table 1). Our criteria for selecting loci were rooted in taxonomic coverage (suitable for a wide range of taxa), taxonomic resolution (species identification) and fragment size. Small fragments not only accommodate the short length of Illumina reads (up to 250 bp) but also allow for uniform sizing across amplicon targets, enabling multiplexed PCR reactions under some conditions. For vector taxonomic identification, we selected two widely recognised amplicons targeting the second internal transcribed spacer (*ITS2*) (Batovska et al. 2016) and the mitochondrial cytochrome oxidase subunit I (*COX1*) (Zhang and Hewitt 1997). To identify the source of blood meals, we tested the extensively used mitochondrial *16S* ribosomal RNA amplicon (Taylor, 1996) and a modified cytochrome b (*cytb*) amplicon (Meyer et al. 1995) based on Kocher et al. (1989). Additionally, we used an amplicon targeting the large subunit of ribulose 1,5‐bisphosphate carboxylase/oxygenase (*rbcL*) (Abbasi et al. 2018) for identification of plant feeding sources, given the presence of plant DNA in nectar and phloem (Leponiemi et al. 2023). For the detection of *Plasmodium spp*. malaria parasites, we assessed the most commonly employed amplicon for the ribosomal *18S* gene (Rougemont et al. 2004). To detect common mutations associated with target‐site insecticide resistance, we amplified the homologous coding regions surrounding two described *Anopheles gambiae* alleles: G119S in acetylcholinesterase‐1 (*ace‐1*), which is linked to resistance against organophosphates and carbamates (Weill et al. 2004), and L1014S in voltage‐gated sodium channel (*vgsc*), which is suggested to confer resistance to pyrethroids and DDT (Lol et al. 2019).

**TABLE 1:** List of primers with Nextera adapters (underlined) appended.

Gene	Round one primer with Nextera adapters appended sequences	Reference
***ITS2*** Forward (5′ > 3′) Reverse (5′ > 3′)	TCGTCGGCAGCGTCAGATGTGTATAAGAGACAGGCTCGTGGATCGATGAAGAC GTCTCGTGGGCTCGGAGATGTGTATAAGAGACAGTGCTTAAATTTAGGGGGTGTAGTCAC	Batovska et al. (2016)
***COX1*** Forward (5′ > 3′) Reverse (5′ > 3′)	TCGTCGGCAGCGTCAGATGTGTATAAGAGACAGTACAGTTGGAATAGACGTTGATAC GTCTCGTGGGCTCGGAGATGTGTATAAGAGACAGTCCAATGCACTAATCTGCCATATTA	Zhang and Hewitt (1997)
***16S*** Forward (5′ > 3′) Reverse (5′ > 3′)	TCGTCGGCAGCGTCAGATGTGTATAAGAGACAGCGGTTGGGGTGACCTCGGA GTCTCGTGGGCTCGGAGATGTGTATAAGAGACAGGCTGTTATCCCTAGGGTAACT	Taylor (1996)
***cytb*** Forward (5′ > 3′) Reverse (5′ > 3′)	TCGTCGGCAGCGTCAGATGTGTATAAGAGACAGCCATCCAACATCTCAGCATGATGAAA GTCTCGTGGGCTCGGAGATGTGTATAAGAGACAGGCCCCTCAGAATGATATTTGTCCTCA	Meyer et al. (1995)
***rbcL*** Forward (5′ > 3′) Reverse (5′ > 3′)	TCGTCGGCAGCGTCAGATGTGTATAAGAGACAGTATGTAGCTTAYCCMTTAGACCTTTTTGAAGA GTCTCGTGGGCTCGGAGATGTGTATAAGAGACAGGCTTCGGCACAAAAKARGAARCGGTCTC	Abbasi et al. (2018)
***18S*** Forward (5′ > 3′) Reverse (5′ > 3′)	TCGTCGGCAGCGTCAGATGTGTATAAGAGACAGGTTAAGGGAGTGAAGACGATCAGA GTCTCGTGGGCTCGGAGATGTGTATAAGAGACAGAACCCAAAGACTTTGATTTCTCATAA	Rougemont et al. (2004)
***ace‐1*** Forward (5′ > 3′) Reverse (5′ > 3′)	TCGTCGGCAGCGTCAGATGTGTATAAGAGACAGCCGGGNGCSACYATGTGGAA GTCTCGTGGGCTCGGAGATGTGTATAAGAGACAGACGATMACGTTCTCYTCCGA	Weill et al. (2004)
***vgsc*** Forward1 (5′ > 3′) Reverse 1 (5′ > 3′) Reverse 2 (5′ > 3′)	TCGTCGGCAGCGTCAGATGTGTATAAGAGACAGAGRTGGAAYTTYACNGAYTTY GTCTCGTGGGCTCGGAGATGTGTATAAGAGACAGGTTCGTCTCATTATCC GTCTCGTGGGCTCGGAGATGTGTATAAGAGACAGGCAANGCTAAGAANAGRTTNAG	Lol et al. (2019)

*Note:* Adapters are underlined. R: A/G; Y: C/T; N: A/C/G/T.

### 2.2 DNA Extraction

Both colony and field mosquitoes were subjected to the following DNA extraction method, utilising the entire mosquito rather than just the head or body. We conducted non‐disruptive DNA extractions from individual mosquitoes using the optimised lysis buffer C, prepared with 200 mM Tris pH 8.0, 25 mM EDTA pH 8.0, 0.4 mg/mL proteinase K and 0.05% Tween 20 (Makunin et al. 2022). We prepared a 1:10 dilution of the lysate solution for use as the input template for round one PCR (PCR1). Both the lysate and the lysate dilution were stored at −20°C.

The plants used in the validation experiments section below were subjected to the following DNA extraction method. We sampled leaf tissue using a handheld round hole puncher (5 mm). Three punches were collected and placed in a 1.5 mL microcentrifuge tube. We carried out DNA extractions from the plant tissue using the DNeasy Plant mini kit (Qiagen, USA), following the manufacturer's protocol.

### 2.3 PCR Conditions

We employed a nested PCR protocol to amplify targets for Illumina sequencing. Each forward and reverse primer sequence for the PCR1 was modified by appending a Nextera adapter at the 5′ end (listed on Table 1). As the previously designed primers varied in annealing temperatures, we performed singleplex PCR1 amplifications. The optimised PCR1 reagents and conditions for each DNA barcode primer with the appended Nextera adapter are provided in the S1 file.

We explored various pooling strategies to optimise coverage of amplicons of varying lengths. Pooling of all PCR1 amplicons products from each sample for the subsequent round two PCR (PCR2) resulted in reduced read coverage for the two longest amplicons (*ITS2* and *COX1*; data not shown). As a result, we adjusted our approach for PCR2 indexing and MiSeq runs. The *ITS2* and *COX1* genes were subjected to PCR2 collectively in a single MiSeq run, the *vgsc* gene was indexed and run on the MiSeq individually, and the remaining amplicons (*16S*, *cytb*, *rbcL*, *18S*, *ace‐1*) were indexed collectively and subjected to a separate MiSeq run. Detailed information regarding the PCR2 reaction and the specific thermocycling conditions can be found in the S1 file.

The PCR2 products, whether single or pooled amplicons, were subjected to purification using AMPure XP beads (Beckman Coulter, High Wycombe, UK). We employed an optimised two‐tailed DNA cleanup procedure (described in the S1 file) to eliminate DNA fragments that fell below the expected amplicon size thresholds. We verified the size of each resulting library using the Agilent Bioanalyzer with a High Sensitivity chip, following the manufacturer's protocol. The concentration of the purified amplicon‐pool library was determined via qPCR. This involved a 20 μL reaction containing 10 μL KAPA SYBR FAST qPCR Master Mix (2×), 2 μL Illumina primer premix (10×), 4 μL water and 4 μL of the pooled PCR2 product. We conducted qPCR reactions using the Roche Lightcycler 96 instrument.

For AmpSeq library preparation, we adhered to the protocol of the Illumina MiSeq Reagent Kit v2. The initial library concentration was 6 nM, which was subsequently adjusted to a final loading concentration of 12 pM. To the final library, we introduced a spike‐in of 7.5% PhiX Control. Sequencing was performed on an Illumina MiSeq platform, using paired‐end 250 bp reads. A single‐lane flow cell accommodates pooled amplicon products from up to 192 samples per run. Optimal clustering density occurs between 950 and 1100, resulting in an output of approximately 7–10 Gbp.

### 2.4 Construction and Filtering of Reference Sequence Databases

For the *COX1*, *ITS2*, *cytb*, *16S* and *rbcL* amplicons, we constructed reference sequence databases intended for use in both sequence similarity (BLAST) and phylogenetic placement (Evolutionary Placement Algorithm, EPA) approaches. We sourced *COX1* sequences from the Barcode of Life Data System (BOLD) while *ITS2*, *cytb*, *16S* and *rbcL* sequences were sourced from the National Center for Biotechnology Information (NCBI). We utilised specific keywords such as [*COX1*; mitochondrial; Diptera] to retrieve relevant sequences from NCBI.

Subsequently we carried out a filtration procedure encompassing automated and manual steps. We retained putative reference sequences that exhibited overlap with our amplicon sequence and were not redundant with other sequences of the same taxon. We excluded sequences with unspecific taxa labels, those categorised as “Unverified”, and extinct species. To be conservative, we manually identified and removed anomalous sequences, such as those with a large proportion of ambiguous base calls or unusually high divergence from known close taxonomic relatives. As a final step, we trimmed all sequences to align with the amplicon sequence length, excluding the primer regions for *COX1, cytb* and *rbcL*.

We assessed the taxonomic breadth of the reference databases by retrieving each sequence's entry in the NCBI Taxonomy database, which includes organism names and lineage annotations for every nucleotide sequence of the International Nucleotide Sequence Database Collaboration (INSDC) (Schoch et al. 2020). The curated reference amplicon databases for species typing had representation across order Diptera, with the *COX1* database containing 2859 sequences across 1325 taxa and the *ITS2* database containing 11,608 sequences across 3262 taxa. The curated reference amplicon databases for blood meal sources had representation across clade Tetrapoda, with the *cytb* database containing 25,785 sequences across 6056 taxa and *16S* database at 21,520 sequences across 11,278 taxa. The curated reference amplicon database for nectar meal sources had representation across clade Tracheophyta, with the *rbcL* database containing 27,361 sequences across 24,316 taxa. All databases are available at https://github.com/broadinstitute/amplicon_taxonomic_identification.

### 2.5 Data Processing and Bioinformatic Analysis Pipeline

The data generated by our AmpSeq panel are first demultiplexed per sample using dual indexing barcodes. This process is implemented on the Illumina platform. Per‐sample FASTQ files are obtained, each containing reads from all sets of locus amplicons. Each sample file represents a pool of sequences from either a single amplicon locus, as in the case of the *vgsc* gene, or a group of amplicon loci, as with the *ITS2* and *COX1* genes or the pooled *16S*, *cytb*, *rbcL*, *18S* and *ace‐1* genes. Prior to DADA2 analysis (Callahan et al. 2016), our pipeline performs Nextera adapter removal and quality control and a demultiplexing step using primer sequences to separate amplicon sequence variants (ASVs) from each amplion target within each sample, after which primer sequences are trimmed from ASVs.

Our approach next applies DADA2 independently to each of the amplicon loci followed by the filtering, dereplication, sample inference, chimera identification and merging or concatenation of paired‐end reads from Illumina amplicon data to produce ASVs (Callahan et al. 2016). Depending on the length conservation of the amplicon and sequencing technology read length, the forward and reverse reads will be merged or concatenated. Merging is executed when the sequenced amplicon (paired‐end reads) spans the full length of the PCR product leading to overlap, as observed with *cytb*, *16S*, *rbcL*, *ace*‐1 and *18S* on a MiSeq with 250 bp reads. Alternatively, concatenation is performed when the sequenced amplicon size leads to no overlap in reads, as observed with *ITS2*, *COX1* and *vgsc* on a MiSeq with 250 bp reads. DADA2 produces two output files: a tab‐separated values (.tsv) file containing the sample IDs in the first column and *n* additional columns representing unclassified ASVs identified by DADA2. The cell values signify the read counts per unclassified ASV per sample. The second output is a tab‐separated text file with ASV details, including its base pair sequence (first column) and chimera status (second column).

The next stage of the pipeline employs the DADA2 outputs to remove chimeras and singleton ASVs. To establish a reliable read count threshold for positive detection signals, we first evaluated the read counts from negative controls included in our sequencing runs. Most negative controls showed no reads, but when they did yield reads, we used the maximum read count observed in negative controls as the minimum threshold for subsequent analysis of sample ASVs. ASVs with read counts below 30 or 100, depending on the locus (threshold empirical rationale in S2 file), are discarded on a per‐sample basis to further exclude potential contaminant sequences. The remaining ASVs undergo filtration based on the expected fragment size of each amplicon (Table 2). The amplicon ASVs that successfully pass the post‐DADA2 filtration criteria (including chimera status, minimum read count and expected fragment size) are advanced to the VecTreeID pipeline.

**TABLE 2:** ASV filtration criteria post‐DADA2 per amplicon. Fragment size in base pairs after read merging/concatenation and primer trimming, as well as analysis criteria, are listed per amplicon.

Amplicon	Expected size (bp)	Minimum read count	Assignment	
			Max haps	Method
*COX1*	643	100	1	EPA Confidence Clade
*ITS2*	318–654	100	2	BLAST Top Hit
*cytb*	308	30	All	EPA Confidence Clade
*16S*	91–102	30	All	BLAST Top Hit
*rbcL*	313–452	30	All	EPA Confidence Clade
*18S*	107–117	30	All	BLAST Top Hit
*ace‐1*	154	50	2	k‐mer Genotyping
*vgsc*	209–268	50	2	k‐mer Genotyping

Abbreviations: bp, base pair; Max Haps, maximum number of haplotypes assigned.

Our vector‐focused AmpSeq bioinformatic pipeline, VecTreeID, is composed of a series of customised scripts (S3 file—Bioinformatics Methods Supplement). The pipeline checks ASVs against the curated reference database for similarity in order to pass an artefact filter. ASVs are ultimately assigned to a taxon if the best database hit passes the identity threshold using the BLAST Top Hit metric, or if placed in an EPA Confidence Clade (the phylogenetic clade containing all plausible matches) for loci amenable to phylogenetic placement (Figure 1). All scripts are available at https://github.com/broadinstitute/amplicon_taxonomic_identification.

Filtered ASVs undergo either taxonomic identification (*COX1*, *ITS2*, *cytb*, *16S* and *rbcL*) or k‐mer based genotyping (*ace‐1* and *vgsc*). For taxonomic identification, the pipeline initially identifies an ASV's BLAST Top Hit, which we retained for analysis if it passed the pipeline's default minimum criteria of 95% coverage and 97% identity (Supporting File S3). Additionally, three amplicons (*COX1*, *cytb* and *rbcL*) are sufficiently conserved across taxa to enable further taxonomic analysis via phylogenetic placement with EPA‐ng (Barbera et al. 2019). For phylogenetic placement, VecTreeID first constructs a phylogenetic reference tree (RT) for each amplicon locus employing the same reference database used in the BLAST approach. These RTs span the spectrum of taxonomic diversity we expect to sample and are generated using the maximum likelihood method within FastTree, employing the ‐m GTRCAT model (Price, 2010). In brief, the EPA‐ng placement procedure commences by introducing the ASV onto a branch of the phylogenetic RT, creating a new tip. The likelihood score of the resultant new tree is subsequently re‐evaluated. This process iterates, with the ASV being successively relocated to other branches. In order to assess the confidence of the phylogenetic ASV placement, the branch probability is computed and reported as likelihood‐weight‐ratio (LWR) values onto branches. An LWR value close to 1 is indicative of robust support for the placement. In situations where reference sequence information is lacking or phylogenetic resolution is poor, the algorithm can yield multiple placements with low LWRs dispersed throughout the tree. After placement with EPA‐ng, a phylogenetic assignment is made via a confidence clade. The confidence clade encompasses all placements whose combined LWRs pass the confidence clade threshold. The confidence clade may consist of one or several species, any of which are considered a plausible identity of the ASV. If the BLAST assignment conflicts with the phylogenetic outcomes, manual examination of the ASV is necessary. Such inconsistency between BLAST and phylogenetic placement might arise because BLAST is a phenetic method and sequence similarity is not necessarily indicative of phylogenetic relatedness (Koski and Golding 2001).

For k‐mer based genotyping, a custom R script is employed to filter the DADA2 output, identifying, at most, the top two ASVs per sample that pass our minimum read threshold and other filters. This is the maximum expected number of ASVs for diploid nuclear loci like *ace‐1* and *vgsc*. Following the identification of the top two ASVs based on their read counts, an unaligned fasta file is generated and used downstream. Our approach to variant calling for *ace‐1* and *vgsc* genes leverages a pre‐established table of k‐mers categorised by whether they contain the resistance‐associated nucleotide at the target site, while acknowledging that not all k‐mers have been tested for resistance effects in all species. An unaligned ASV is classified as (potentially) resistant or susceptible based on exact matches to this table, or else it yields a result of “no kmer found”. The k‐mer table can be modified to incorporate new sequences. Consequently, the pipeline produces a table indicating the ASV, its classification as either resistant or susceptible, and subsequently associates this information with each sample. The final report connects each sample ID to the resistant or susceptible status for both *ace‐1* and *vgsc* genes.

### 2.6 Evaluating Taxonomic Breadth, Specificity and Sensitivity

We assessed the taxonomic breadth of each amplicon by successfully amplifying representative taxa falling within their respective taxonomic detection range, detailed in the S4 file. Amplicon specificity was evaluated for any cross‐reactions outside their expected taxonomic detection range. Sensitivity for each assay was tested using a serial dilution of DNA template ranging from 1 to 0.001 ng/μL.

For *ITS2*, *COX1*, *ace‐1* and *vgsc* genes, the taxonomic breadth was evaluated using male mosquitoes from colonies representing five species across three different genera: *Anopheles gambiae* , *Anopheles stephensi*, *Anopheles albimanus* , *Culex pipiens* and *Aedes aegypti* . Taxonomic breadth for *16S* and *cytb* genes was assessed using commercial genomic DNA from human, dog, chicken and pig. In the case of the *rbcL* gene, genomic DNA from 10 plant species was used, representing 10 different angiosperm orders: *Mimosa pudica* (Fabales: Fabaceae), *Epiphyllum phyllantus* (Caryophyllales: Cactaceae), *Cattleya percivaliana* (Asparagales: Orchidaceae), *Aechmea melinonii* (Poales: Bromeliaceae), *Monstera deliciosa* (Alismatales: Araceae), *Persea americana* (Laurales: Lauraceae), *Aristolochia gigantea* (Piperales: Aristolochiaceae), *Passiflora edulis* (Malpighiales: Passifloraceae), *Heliconia rostrata* (Zingiberales: Heliconiaceae) and *Psychotria viridis* (Gentianales: Rubiaceae). To assess *rbcL* sensitivity, a serial dilution of the DNA template ranging from 1 to 0.001 ng/μL from 2 out of the 10 species were used ( *M. pudica* and *M. deliciosa* ). These genomic DNAs were used not only to assess any cross‐detection among amplicons, but also to calibrate our VecTreeID pipeline and evaluate their accuracy identifying the mosquito, blood meal and plant meal species. Male insectary mosquitoes (fed on sucrose solution) were used as biological negative controls for meal source experiments (*16S*, *cytb* and *rbcL*). All sensitivity experiments were prepared and run as a single pool on the MiSeq platform to mitigate discrepancies in amplicon read counts associated with technical variation in DNA library preparation. Sequencing coverage (read count) was used as a metric for evaluating sensitivity.

### 2.7 Blood Meal Detection Time Series

To determine persistence of the blood meal genetic signal inside the mosquito digestive system over time, we fed female *An. gambiae* with human blood. Ten engorged females were harvested at zero, 12, 24, 36, 48, 54, 60, 66, 72, 78 and 84 h post‐blood feeding for analysis. The mosquitoes were reared in laboratory cages at a temperature of 27°C, with a relative humidity ranging from 70% to 80% and 12‐h light–dark photoperiod.

### 2.8 Comparative Analysis of PCR Success and Sensitivity in *Plasmodium* Detection

In an attempt to reduce laboratory effort and costs, we assessed the effectiveness of using lysate extract in comparison to purified DNA as template for PCR1 reactions. We also evaluated the ability to detect parasite infection when employing a non‐disruptive DNA extraction method (enzymatic lysis). For these two purposes, *An. gambiae* colony mosquitoes infected with *Plasmodium falciparum* were used. The post‐infection time points that were sampled included seven, 13, 14 and 20 days. Mosquitoes underwent the non‐disruptive lysis protocol. As detailed earlier, 60 μL of lysate solution was prepared per individual mosquito, but only 40–50 μL was recovered due to some evaporation. From the 40–50 μL of lysate, a 1:10 lysate dilution was prepared. The remaining lysate was used to carry out DNA purification using a DNeasy Blood & Tissue kit (Qiagen, USA), following the manufacturer's specifications. Both the diluted lysate and purified DNA were employed as template for PCR1. At each time point, five to six mosquitoes were sampled, and three replicates were performed per mosquito and DNA extraction method.

For the experiments involving *Plasmodium* DNA, we did not use parasite‐infected mosquitoes. Instead, we utilised an aliquot containing approximately 1 × 10^6^ sporozoite cells. DNA extraction was performed using a HighPrep Blood & Tissue DNA Kit (MagBio, USA), following the manufacturer's specifications for cultured cells. The final elution volume was 50 μL, leading to an estimated concentration of 20,000 copies/uL.

### 2.9 Multiple Meal Detection

To assess the capability of detecting multiple blood and nectar meal sources from individual specimens, two types of experiments were conducted, each comprising four mixtures. In the first experiment, genomic DNA from species “A” was combined with species “B” in a one‐to‐one ratio. Four “one‐to‐one” mixtures were evaluated, representing a DNA gradient. These mixtures were as follows (Species A:B): 1:1, 0.1:0.1, 0.01:0.01 and 0.001:0.001 ng/μL. The second experiment consisted of three mixtures in which one of the species was maintained at a high and constant concentration (1 ng/μL), while the second species was present at progressively lower DNA concentrations (0.1, 0.01 and 0.001 ng/μL).

To validate the detectability of multiple blood meals, commercial genomic DNA from chicken and human was utilised. To validate the detectability of multiple nectar meals, genomic DNA from *M. pudica* and *M. deliciosa* was experimentally extracted in the laboratory. All DNA mixtures were prepared under mosquito male lysate background, and three replicates of each DNA mixture were processed. Library preparation and sequencing were carried out as described before.

### 2.10 Field Mosquito Specimens

A total of 1986 field‐collected mosquitoes from Guyana, Venezuela and Colombia were analysed using the complete panel (Table S1). Amplicons from the 1986 mosquito samples were processed in 26 MiSeq runs, as we experimented with different pooling and multiplexing approaches.

## 3 Results

### 3.1 Blood Meal Detection Time Series

**FIGURE 2:**
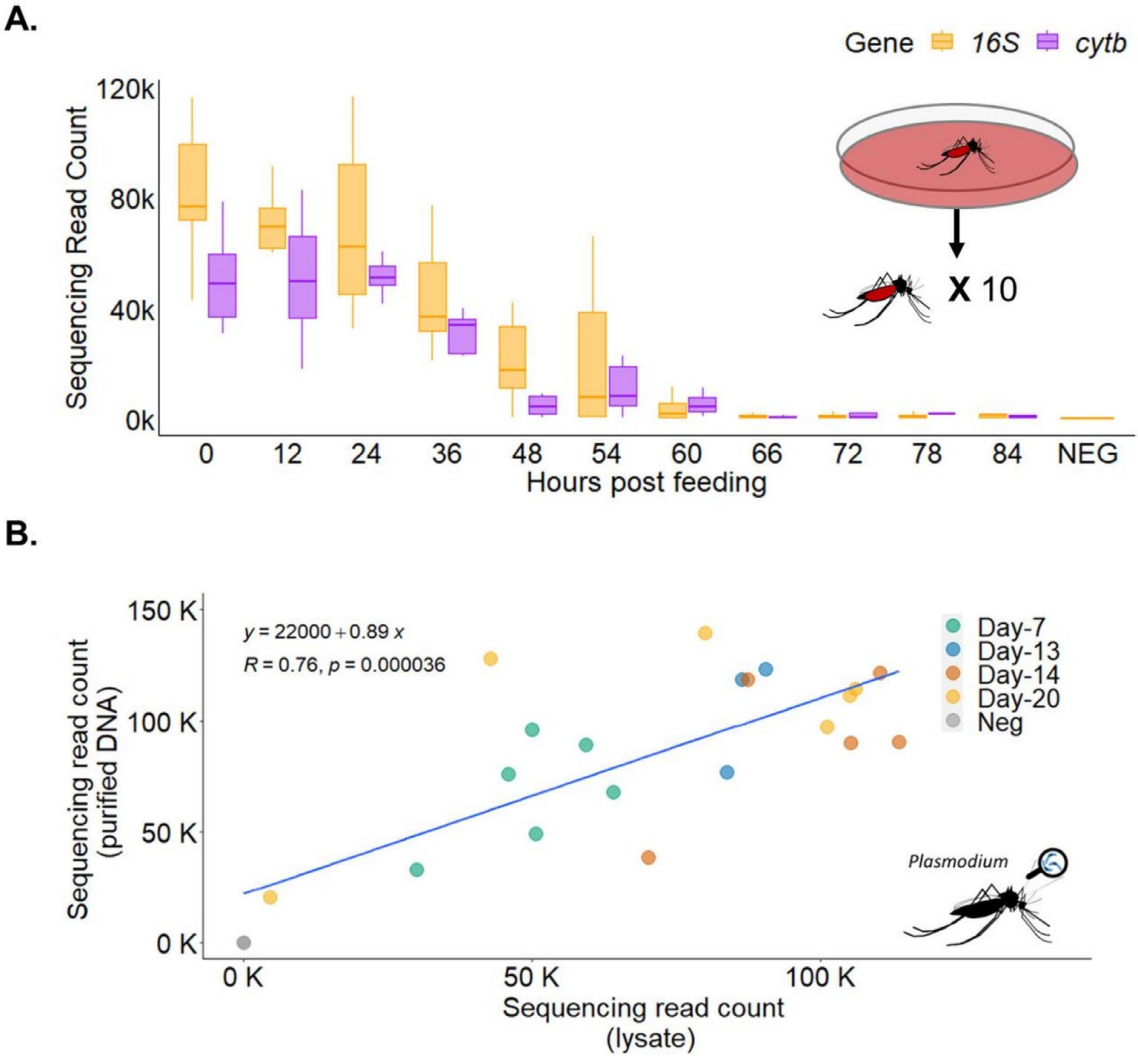
Time series for blood‐meal and parasite detection. (A) Blood Meal DNA is reliably detected at least 36‐h post‐feeding with both *16S* and *cytb* amplicons. Ten mosquitoes were sampled at 0, 12, 24, 36, 48, 54, 60, 66, 72, 78, 84 h post‐feeding. Extracted DNA was used as PCR1 template. Blood meal signal is measured as sequencing read counts; (B) Detection of *P. falciparum* DNA in infected mosquitoes is very similar regardless of whether purified DNA or diluted lysate is used as PCR1 template. Infected mosquitoes were sampled at Day 7, 13, 14 and 20 post‐infection. NEG, No template negative control.

Both the *16S* and *cytb* amplicons produced blood meal signals that decreased over the post‐feeding period. All 10 fed mosquitoes exhibited blood meal signals from 0 to 36 h post‐feeding, and 5 out of 10 exhibited blood meal signals beyond 36 h (Figure 2A), suggesting this assay should reliably detect most recent blood meals in field‐collected specimens.

### 3.2 Lysate vs. Purified DNA as Template for PCR Experiment

Diluted lysate from the non‐disruptive DNA extraction method proved to be as effective as purified DNA template for round one PCR. Both diluted lysate and purified DNA extraction yielded high‐quality sequencing data based on read counts. Our VecTreeID pipeline successfully confirmed the identities of the mosquito and parasite as *An. gambiae* and *P. falciparum*, respectively. Throughout the evaluated post‐infection period, the parasite signal was detectable as early as the seventh day and persisted up to 20 days post‐infection (Figure 2B).

### 3.3 Taxonomic Breadth, Specificity and Sensitivity

**FIGURE 3:**
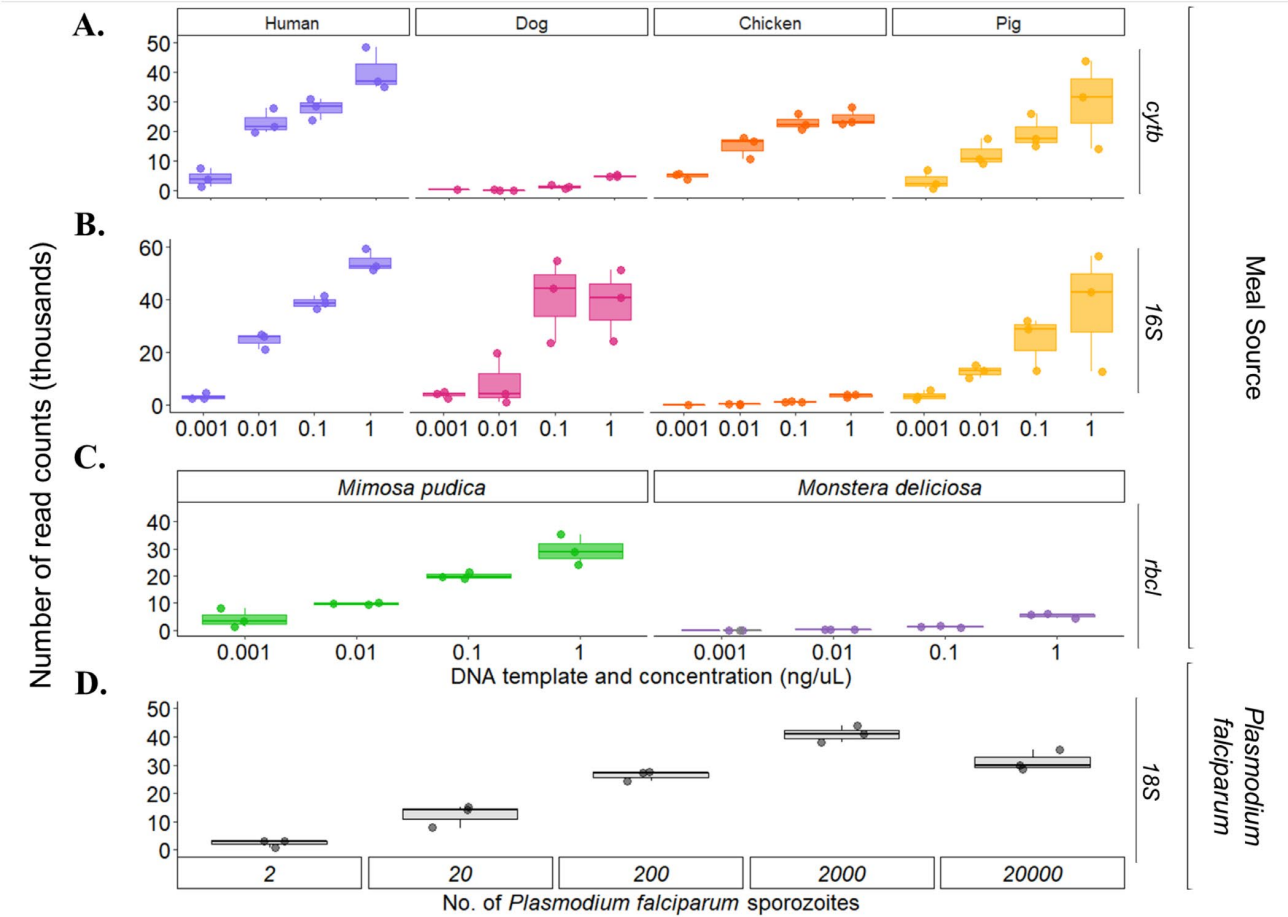
Sensitivity and taxonomic breadth validation of meal source and parasite detection amplicons. Taxonomic breadth and limits of detection were assessed with a DNA concentration gradient made from genomic DNA from 4 vertebrate species and 10 plant species representing 10 families (only two assays are shown). Parasite detection was assessed using a gradient of sporozoite counts. (A) *cytb*; (B) *16S*; (C) *rbcL*; (D) *18S* amplicon.

The *ITS2* and *COX1* amplicons amplified effectively and produced high‐quality sequencing reads successfully for all five tested mosquito species: *An. gambiae*, *An. stephensi*, *An. albimanus*, *C. pipiens* and *A. aegypti* . Similarly, both the *16S* and *cytb* amplicons yielded robust amplification and sequencing data for the four vertebrate species tested: human ( *Homo sapiens* ), dog ( *Canis familiaris* ), chicken ( *Gallus gallus* ) and pig ( *Sus scrofa* ). The sensitivity varied by gene (Figure 3A,B). The *16S* amplicon exhibited lower sensitivity when detecting chicken in comparison to *cytb*, while *cytb* showed lower sensitivity when detecting dog. This PCR bias is likely due to species‐specific genetic variation at the primer binding sites, which can impact amplification efficiency. These differences highlight the complementarity of using multiple markers for detecting a phylogenetically broader range of blood meal sources. Similarly, the *rbcL* amplicon successfully amplified the 10 plant species with variable sensitivity (Figure 3C and S4 File‐Figure 2) While the performance of the *18S* amplicon was evaluated in a single *Plasmodium* species, it demonstrated a detection range from 2 to 20,000 sporozoites.

### 3.4 Detection and Discrimination of Multiple Meal Sources

**FIGURE 4:**
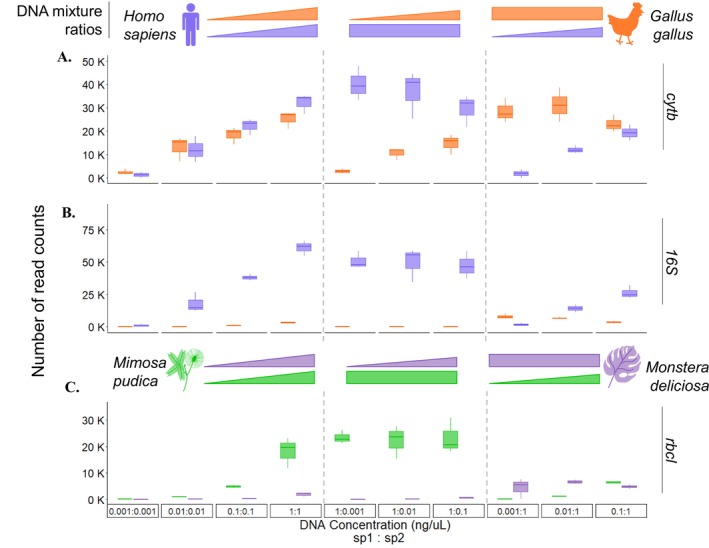
Assessment of mock DNA mixtures to validate sensitivity for detecting multiple meal sources. The left panel shows a one‐to‐one ratio mixture of Species 1 and 2 at varying DNA concentrations. The middle panel shows Species 2 DNA at gradient concentrations (1, 0.1, 0.01 and 0.001 ng/μL) mixed with a constant concentration of Species 1 DNA (1 ng/μL). The right panel reverses the middle panel setup. All DNA mixtures used male mosquito lysate as background and three PCR replicates were run for each DNA mixture. Amplicons tested were (A) *cytb*; (B) *16S* and (C) *rbcL*, with detection signal based on sequencing read counts (thousands).

We successfully identified and differentiated individual components within mixed meal sources across all prepared mixtures (Figure 4). In the first “one‐to‐one” experiment mixtures, the *cytb* gene generated comparable read counts for each DNA template in the mixture (Figure 4A left panel). Conversely, the *16S* and *rbcL* genes exhibited variations in read count yields for each DNA template in the mixtures, further supporting the PCR bias observed in our sensitivity experiments (Figure 4B,C left panel).

In the second experiment mixtures, the *cytb* gene produced the anticipated detection signal based on the mixed ratios. Specifically, when the species at a higher constant DNA concentration generated greater number of read counts, the second species with a lower DNA concentration yielded fewer read counts in comparison to the first species (Figure 4A middle panel). This behaviour was similarly observed in the reciprocal experiment for the *cytb* gene (Figure 4A right panel).

For amplicons exhibiting taxonomic bias in PCR amplifications, such as *16S* and *rbcL*, DNA from the species with stronger affinity was favoured over DNA with weaker affinity (Figure 4B,C middle panel), irrespective of DNA concentration. However, for both amplicons, the DNA with lower affinity still yielded a sufficient number of high‐quality reads, enabling identification of the respective blood source when present. In the reciprocal case of the second experiment for *16S* and *rbcL* amplicons, overall read counts were slightly lower, yet adequate high‐quality reads remained available for identifying each present source (Figure 4B,C right panel).

### 3.5 Field Mosquito Specimens

We analysed a total of 1986 field mosquitoes from Colombia, Guyana and Venezuela using the VecTreeID pipeline, detailed in the S3 File. Mosquito taxonomic assignments are reported from the species level to above genus level. Additionally, we included a category for taxa with no taxonomic assignment for cases where no successful PCR product was obtained (indicated by zero reads retrieved from all amplicons) or cases of potential cross‐sample contamination (such as incongruency in genus level identification among markers).

**FIGURE 5:**
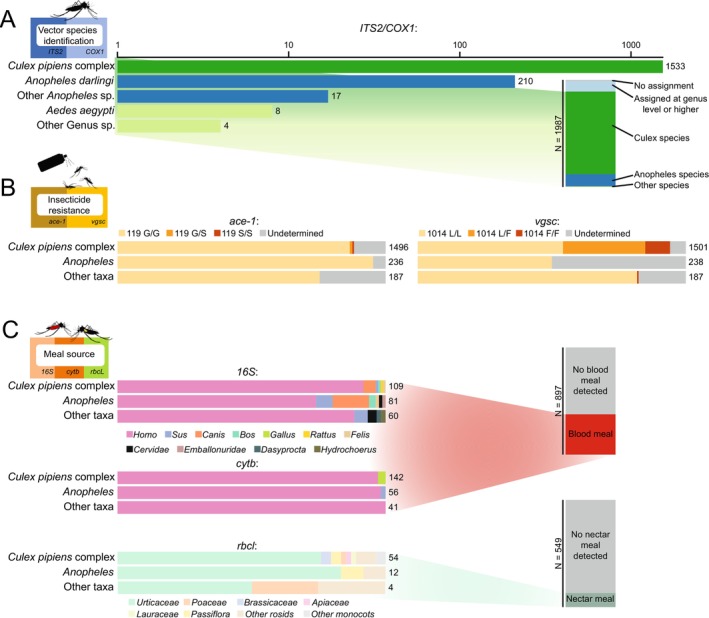
VecTreeID results for field samples. A total of 1986 mosquitoes from Colombia, Guyana and Venezuela were examined, although not all were amplified at all loci. (A) Vector taxonomic identification with *COX1* and *ITS2*. Most specimens could be identified conclusively, of which 99% were either *Culex pipiens* complex or *Anopheles* species. (B) Insecticide resistance assessment with *ace‐1* and *vgsc*. Putative resistance genotypes (S‐ at *ace‐1* and F‐ at *vgsc*; not phenotypically validated) were observed at both loci, especially for *Culex pipiens* complex mosquitoes. (C) Meal source identification with *16S*, *cytb* and *rbcL*. Blood meals from various vertebrates, mostly human, were detected in 337 mosquitoes. Nectar meals from various plant families, mostly *Urticaceae*, were detected in 72 mosquitoes.

The VecTreeID pipeline demonstrated high effectiveness, with 99% of the tested mosquitoes yielding a taxonomic assignment (1966/1986) (Figure 5A). Among the successfully identified mosquitoes, 78% were classified as *Culex pipiens* complex (1533/1966), 11% as *Anopheles darlingi* (210/1966), 1% as other anophelines (including *An. marajoara*, *An. costai*, *An. intermedius*, *An. konderi, An. nuneztovari* and *An. triannulatus*) (17/1966), less than 1% as *Aedes aegypti* (8/1966), and less than 1% as other species from the *Aedeomyia*, *Coquillettidia*, *Mansonia* and *Culex* genera (4/1966) (Figure 5A and Table S1).

For the insecticide resistance amplicons, 1.6% of the *Culex pipiens* complex mosquitoes (24/1496) carried at least one nonsynonymous allele associated with resistance in the *ace‐1* gene (G/S or S/S at the homologous position for *An. gambiae* codon 119) (Weill et al. 2004) (Figure 5B). In contrast, only alleles associated with susceptibility at this codon position were found in *Anopheles* species and other non‐*Culex* taxa. Meanwhile, 40% of the *Culex pipiens* complex mosquitoes (600/1501) carried at least one nonsynonymous allele associated with resistance in the *vgsc* gene (L/F or F/F at the homologous position for *An. gambiae* codon 1014) (Lol et al. 2019). In contrast, only alleles associated with susceptibility at codon position 1014 in the *vgsc* gene were present in *Anopheles* species (Figure 5B).

Blood meal detection was observed in 37.6% (337 / 897) of the mosquitoes sequenced at *cytb* and *16S* (Figure 5C). *Culex pipiens* complex mosquitoes fed on humans ( *Homo sapiens* : 138 instances) and chickens (*Gallus*: 3 instances) according to the *cytb* locus. *Culex pipiens* complex mosquitoes fed on humans ( *Homo sapiens* : 100 instances), dogs (*Canis*: 5 instances), cow (*Bos*), rat (*Rattus*), cat (*Felis*) and a pig (*Sus*) according to the *16S* locus. *Anopheles darlingi* mosquitoes fed on humans ( *Homo sapiens* : 49 instances) according to the *cytb* locus. *Anopheles darlingi* mosquitoes fed on humans ( *Homo sapiens* : 54 instances), dogs (*Canis*: 9 instances), pigs (*Sus*: 3 instances), cows (*Bos*: 2 instances), cat (*Felis*), deer (Cervidae) and a bat (Emballonuridae) according to the *16S* locus. Specimens representing the remaining taxa had primarily fed on humans, but there were also detected instances of feeding on South American endemic species such as a capybara ( *Hydrochoerus hydrochaeris* ), pudu ( *Pudu puda* ), brocket deer ( *Mazama americana* ) and an agouti ( *Dasyprocta punctata* ). Furthermore, the *cytb* and *16S* amplicons showed concordance primarily in human meal sources but demonstrated complementarity in the detection of other blood meals, as shown in the combined meal source detection sensitivity experiments. For example, chicken was detected with *cytb* but under detected by the *16S* amplicon, whereas dog was detected by *16S* but not by *cytb* (Figure 5C).

Nectar meal detection was observed in 13.1% (72/549) of the mosquitoes sequenced at *rbcL* (Figure 5D). *Culex pipiens* complex mosquitoes exhibited a diverse range of plant feeding sources, including families such as Urticaceae, Poaceae, Lauraceae, Brassicaceae and Fagaceae. *Anopheles* species showed plant intakes from Urticaceae, while other taxa fed on plants from Urticaceae, Poaceae and Malpighiaceae. *Plasmodium* detection was conducted exclusively on *Anopheles* species, with no *Plasmodium* detected in any of the tested anopheline mosquitoes.

## 4 Discussion

We have demonstrated the utility of an Illumina‐based amplicon panel, coupled with the VecTreeID pipeline for comprehensive mosquito surveillance and vector control applications. Illumina‐based implementations of previously developed barcoding amplicons can provide vector taxonomic identification, as well as determine sources of blood and nectar meals, *Plasmodium* infection status, and target‐site insecticide resistance genotypes. Our study demonstrated that a single amplicon sequencing run can successfully identify mosquito species from at least 334 mosquitoes (*ITS2*‐*COX1*). A second amplicon sequencing run can reveal blood meal sources (*16S*‐*cytb*), nectar meal sources (*rbcL*) and *Plasmodium* infection (*18S*) from at least 192 mosquitoes. Additionally, a third amplicon sequencing run can identify mutations associated with a second insecticide resistance locus (*vgsc*) from at least 334 mosquitoes. This comprehensive approach demonstrates the capability to analyse various crucial factors across multiple runs. While Makunin et al. (2022) demonstrated a high‐throughput method capable of processing over 1000 mosquitoes per MiSeq run, our method offers detailed and specific insights into multiple aspects of mosquito biology and vector competence. Based on our experience, processing 1000 samples across eight amplicons, each requiring PCR1 with an average of 2.75 h per plate, would take approximately 7.5 days to complete under an 8‐h workday schedule using four PCR machines. This timeframe will increase if fewer PCR machines are available. For PCR2, PCR1 products are pooled by sample for indexing, following our pooling strategy: *ITS2* and *COX1* amplicons were pooled, the *vgsc* gene was indexed and sequenced individually, while *16S*, *cytB*, *rbcL*, *18S* and *ace‐1* amplicons were pooled and run in a single MiSeq (S1). Preparation for PCR2 and amplicon libraries, along with sequencing, adds another week, with sequencing runs typically requiring 24 h. Thus, within a 2‐week period, a facility can generate genotypic data for approximately 1000 mosquitoes across all targeted amplicons. This approach enhances the ability to conduct comprehensive and targeted surveillance, potentially leading to a more nuanced understanding of mosquito populations and more effective vector control strategies.

This AmpSeq panel, when paired with the VecTreeID pipeline, enables broad identification across a wide taxonomic range of dipteran vectors using a single laboratory and informatic protocol. Successfully amplified genera include *Anopheles*, *Culex*, *Aedes, Aedeomyia, Coquilletidia, Mansonia* and *Chrysotus*. Both *COX1* and *ITS2* are established markers in insect barcoding, known for their utility but also their limitations such as poorly characterised intraspecific variability, shared haplotypes between species, and discrepancies with morphology or other molecular markers (Marrelli et al. 2006; Meier et al. 2006). Because our Illumina‐based approach sequences these loci less comprehensively than traditional Sanger sequencing, it identifies fewer informative sites, potentially exacerbating these challenges. Thus, especially within species complexes, comprehensive vector identification may necessitate additional loci to achieve higher taxonomic resolution for specific taxa of interest (Makunin et al. 2022). Our contribution lies not in developing the *COX1* and *ITS2* primers—already widely used across diverse vector genes—but in demonstrating their adaptability for use in an Illumina‐based pipeline without compromising their ability to amplify a broad taxonomic range. These amplicons may be further adaptable to long‐read sequencing platforms for setting with access to those technologies, and long reads would allow full sequencing of longer and more informative amplicons, but would require redevelopment of amplification protocols as well as PCR/sequencing error filtration approaches to accommodate the distinct error modes of long‐read sequencing.

Our approach can successfully identify single and multiple feeding sources with high consistency, accuracy, specificity and sensitivity as shown in Figures 3 and 4. Consistency was shown by our feeding experiment which showed successful identification of a blood source within 36 h. Our *rbcL* primers amplify all 10 tested plant orders (S4 File‐Figure 2) as well as additional orders detected in field samples (e.g. Rosales, Fagales, Myrtales), suggesting broad‐spectrum activity. Although our AmpSeq feeding profile panel shows differential amplicon yields at different DNA concentrations and when multiple DNA templates are present, in all cases this differential PCR yield does not hamper the ability to detect and identify the meal source, highlighting the accuracy, specificity and sensitivity of each assay. Importantly, our field collected mosquitoes fed on multiple hosts, a fact that can provide insights about host attraction, biting and resting behaviour when experimental design is planned accordingly (Diouf et al. 2021). Our data show that both *An. darlingi* and *C. pipiens* exhibit anthropophilic behaviour and share a strong affinity for *Urticaceae* (plausibly *Cecropia*) nectar, and suggest that *An. darlingi* feeds on a broader range of non‐human hosts and plants relative to *C. pipiens* (Figure 5C). In other studies, the proportion of mosquitoes with PCR‐detectable plant DNA can be high (e.g. 42% of *An. sergentii* in Israel, Junnila et al. 2010) while estimates of nectar feeding based on non‐molecular methods can be quite low (e.g. 0.06% from visual inspection of abdomens among Canadian samples, (Cassone et al. 2024). While we here refer to plant DNA as nectar for readability, we note that mosquitoes also ingest other plant tissues, and feeding habits vary seasonally and among species and ecosystems. Our detection rate of nectar meals is consistent with the wide range of plausible rates suggested by previous observations. With respect to *Plasmodium* mosquito infection, our time series experiment shows consistency and sensitivity of the assay from Days 7–20 (Figure 2B). Infected Anopheles typically harbour > 1000 sporozoites in their salivary glands, well withing the sensitivity of our assay (Andolina et al. 2024). The detection of *Plasmodium* over this period provides vital information regarding the life cycle of the parasite in its vector and its potential for transmission (Wang et al. 2018). It is important to note that our sample preparation involves using the whole mosquito rather than dissecting only the head and thorax (Kumpitak et al. 2021). This approach does not distinguish whether *Plasmodium* detection is due to sporozoites in the mosquito's salivary glands or from potentially infected ingested blood containing gametocytes. However, this distinction can be resolved by dissecting the mosquito before lysis, thereby differentiating an infected blood meal from sporozoite carriage. Despite *An. darlingi* being a principal malaria vector in the Americas, our field mosquito samples did not exhibit any *Plasmodium* infections, which aligns with the typically low rates of *Plasmodium* detection in field‐collected anophelines mosquitoes (e.g. 0.5%, Sukkanon et al. 2022).

We detected a large proportion of *Culex pipiens* complex mosquitoes with putative insecticide resistance genotypes, a potentially consequential finding suggesting widespread adaptation to anthropogenic xenobiotics (Figure 5B). However, target site insecticide resistance polymorphisms should be interpreted with caution for polymorphisms not yet phenotypically validated. Furthermore, *ace‐1* and *vgsc* are just two of several important insecticide‐resistance genes that also include cytochrome P450s, glutathione S‐transferases, *rdl* and others (Fouet et al. 2018). Thus, a negative amplicon result cannot be taken as proof of insecticide susceptibility.

**FIGURE 6:**
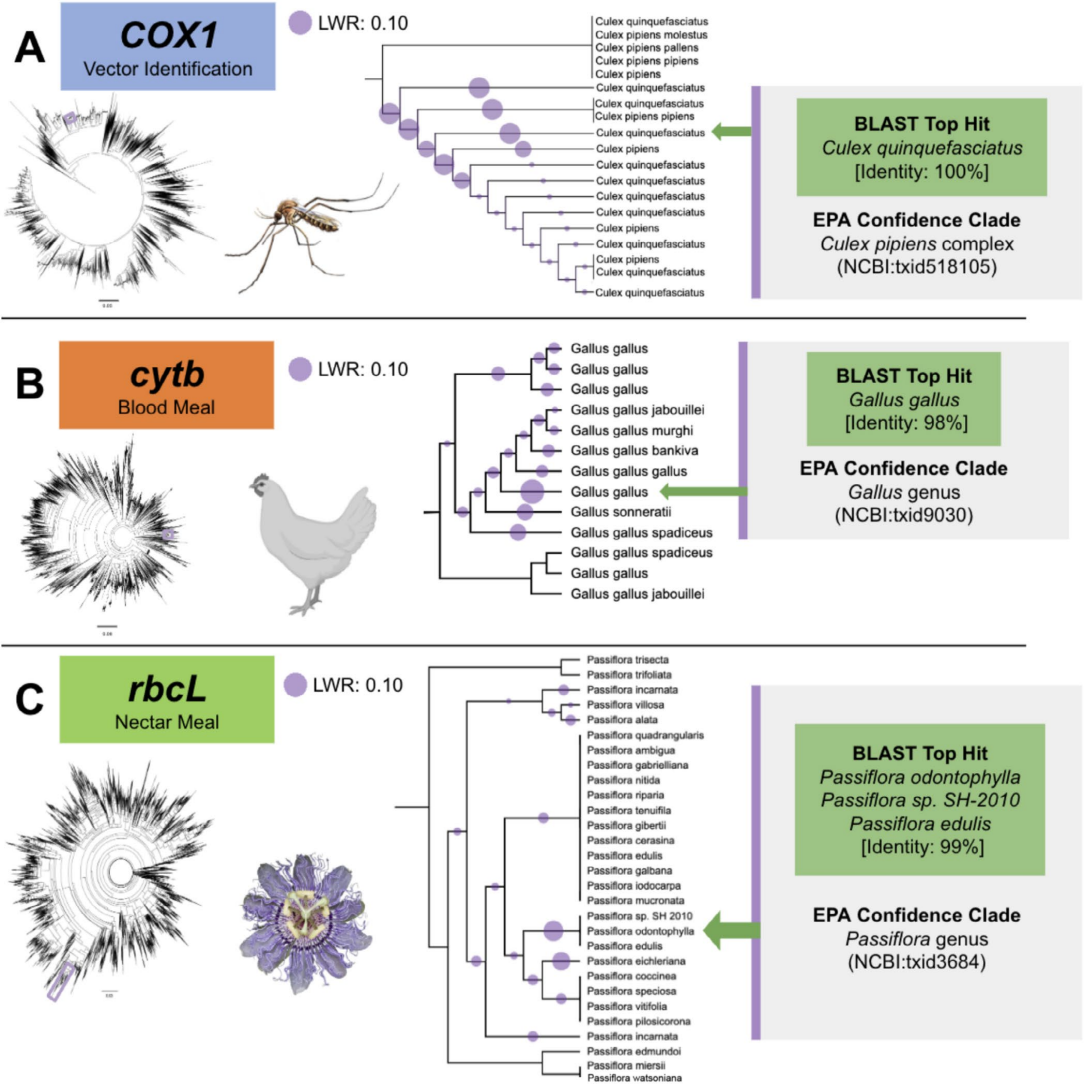
Phylogenetic placement using VecTreeID for taxonomy assignment of three representative ASVs from our field samples. Each cladogram shows a portion of the maximum likelihood phylogeny from reference amplicon sequences with EPA‐ng placements as purple circles sized by Likelihood Weight Ratio. The box highlights the taxonomic assignment for each placement (green arrow: BLAST Top Hit, purple bar: EPA confidence clade). (A) Placement of *COX1* ASV to the *Culex pipiens* complex. (B) Placement of *cytb* ASV to the *Gallus* genus. (C) Placement of *rbcL* ASV to the *Passiflora* genus.

A major goal of our approach has been to accommodate uncertainty and minimise overconfident misidentification. Low‐coverage erroneous ASVs can be generated via a variety of means including sequencing error, PCR‐mediated recombination, index hopping, or sample contamination; thus our pipeline only considers the top ASVs and features robust criteria for ASV inclusion. In addition, the best match in a database by raw sequence similarity does not necessarily represent the taxonomic identity of a sample, and in our experience is sometimes ecologically implausible (e.g. a species not found in that habitat). Our tree‐based analysis accounts for this uncertainty and reports a higher‐level taxonomic clade when a single species cannot be assigned with high confidence (Figure 6).

A limitation of our current approach is its reliance on reference sequence databases, which may lack representation of diverse taxa, especially in biodiverse, underrepresented regions. This limitation could mean that negative results indicate either a true absence of a blood or nectar meal or, alternatively, the absence of the species' genetic information in existing databases. This ambiguity is particularly relevant in high‐biodiversity areas, such as rural and remote regions, where many local plant and animal species remain unsequenced. To mitigate this, we incorporated phylogenetic placement analysis (Figure 6), which improves identification accuracy by clustering unknown sequences with phylogenetically related taxa. However, ambiguous results can still arise if the database lacks sequences closely related to the unidentified taxon. Future enhancements could involve expanding local reference libraries and incorporating additional loci to maximise confidence in taxonomic assignment, particularly for species complexes of local importance.

Furthermore, in our manually curated reference databases we have identified and removed potentially erroneous reference sequences with low sequence similarity to known close relatives. In our view, this cautious approach is preferable to an overly specific but possibly incorrect species assignment. Assignment errors are known to occur (Cheng et al. 2023) and we suspect they could account for certain unexpected results occasionally reported in barcoding studies.

The vector specific indicators presented in this study represent a maturation of entomological surveillance tools with demonstrated utility, enabling their systematic application to large sample collections. Surveillance programs will benefit from implementing this molecular and bioinformatic toolkit as data‐driven research to tailor effective control interventions and reduce the transmission of vector‐borne diseases. Future iterations of the panel could include markers for additional vector‐borne parasites and pathogens, as well as additional loci to maximise confidence in taxonomic assignment or resolve species complexes of local importance. Despite early suspicions that whole genome shotgun sequencing would render molecular barcoding obsolete (Taylor and Harris 2012), we have shown that a carefully calibrated Illumina‐based panel can provide cost‐effective utility, especially in the realm of public health.

## Author Contributions

Raquel Lima‐Cordón, Jason T Mohabir, Angela M Early, Horace Cox, Socrates Herrera, Margaret Laws conceptualised the research, formulating and evolving the overarching research goals and aims. Raquel Lima‐Cordón, Jason T Mohabir, Mohini Sooklall, Maria Eugenia Grillet, Martha Quinones were responsible for data curation, including annotating, scrubbing and maintaining research data. Raquel Lima‐Cordón, Jason T Mohabir, Aina Martinez Zurita, Ruchit Panchal conducted formal analysis using statistical, mathematical and computational techniques. Daniel E Neafsey secured the financial support for the project. Raquel Lima‐Cordón, Meg Shieh, Cheyenne Knox, Sabrina Gobran, Zachary Johnson conducted the investigation, performing experiments and data collection. Jacob A Tennessen, Jason T Mohabir, Angela M Early developed the methodology and created models. Margaret Laws, Daniel E Neafsey managed and coordinated the research activities. Mohini Sooklall, Reza Niles‐Robin, Horace Cox, Maria Eugenia Grillet, Jorge E. Moreno, Socrates Herrera, Martha Quinones provided the necessary resources, including study materials and analysis tools. Jason T Mohabir, Ruchit Panchal, Jacob A Tennessen were responsible for software development and implementation. Daniel E Neafsey, Jacob A Tennessen, Angela M Early supervised the research activities. Raquel Lima‐Cordón, Mohini Sooklall, Cheyenne Knox, Sabrina Gobran, Zachary Johnson validated the results, ensuring replication and reproducibility. Raquel Lima‐Cordón, Jason T Mohabir, Jacob A Tennessen prepared and presented the visualisations. Raquel Lima‐Cordón, Jason T Mohabir wrote the original draft of the manuscript. Raquel Lima‐Cordón, Jason T Mohabir, Mohini Sooklall, Aina Martinez Zurita, Cheyenne Knox, Sabrina Gobran, Zachary Johnson, Margaret Laws, Ruchit Panchal, Reza Niles‐Robin, Horace Cox, Maria Eugenia Grillet, Jorge E. Moreno, Socrates Herrera, Martha Quinones, Angela M Early, Jacob A Tennessen, Daniel E Neafsey reviewed and edited the manuscript, providing critical commentary and revisions.

## Conflicts of Interest

The authors declare no conflicts of interest.

## Supporting information


Data S1.

Table S1.

## Data Availability

All amplicon sequencing data from field samples were submitted to the NCBI Sequence Read Archive (http://www.ncbi.nlm.nih.gov/sra) under accession PRJNA1127569. Software and documentation can be found at https://github.com/broadinstitute/malaria‐amplicon‐pipeline (malaria amplicon pipeline) and https://github.com/broadinstitute/amplicon_taxonomic_identification (VecTreeID).
