# Supplementary material for: A Short‐Read Amplicon Sequencing Protocol and Bioinformatic Pipeline for Ecological Surveillance of Dipteran Disease Vectors
Source: Mol Ecol Resour. 2025 Feb 18;25(6):e14088. doi: 10.1111/1755-0998.14088 (PMC12225702; doi:10.1111/1755-0998.14088)
Supplement: Supplementary file 1 — Data S1. [file MEN-25-e14088-s002.docx]

**Supplemental Information for:**

**A short-read amplicon sequencing protocol and bioinformatic pipeline for ecological surveillance of dipteran disease vectors**

**Authors**: Raquel Lima-Cordón* , Jason T Mohabir* , Mohini Sooklall*, Aina Martinez Zurita, Meg Shieh, Cheyenne Knox, Sabrina Gobran, Zachary Johnson, Margaret Laws, Ruchit Panchal, Reza Niles-Robin, Horace Cox, Maria Eugenia Grillet, Jorge E. Moreno, Socrates Herrera, Martha Quinones, Angela M Early, Jacob A Tennessen, Daniel E Neafsey

* co-first authors

**Table of content**

[**S1 Text: Supplemental wet lab methods** 2](#_Toc182903316)

[1. Mosquito Lysate 2](#_Toc182903317)

[2. Round one PCR (PCR1) Master mix and thermocycler conditions 4](#_Toc182903318)

[3. Round two PCR (PCR2) Master mix and thermocycler conditions 6](#_Toc182903319)

[4. Two-tailed bead clean up. 6](#_Toc182903320)

[**S2 Text: Empirical data informing filtering criteria** 9](#_Toc182903321)

[**S3 Text: Supplemental bioinformatic methods** 10](#_Toc182903322)

[1. Database Curation 10](#_Toc182903323)

[2. Taxonomy Assignment 11](#_Toc182903324)

[**S4 Text: Assessment of taxonomic breadth** 13](#_Toc182903325)

# **S1 Text: Supplemental wet lab methods**

## Mosquito Lysate

We recommend preserving the mosquitoes in 80% or 100% ethanol, as DNA yield is higher than that of non-preserved frozen/dessicated mosquitoes.

Day 1: Plating and Lysing Mosquitoes

**Table 1. Buffer C recipe.** Originally described in Makunin et al. 2022, *Molecular Ecology Resources* (doi: 10.1111/1755-0998.13436). The volumes are provided in µL per mosquito sample and per 96-well plate. For the 96-well, volumes are purposefully overcalculated for 106 samples to accommodate for potential pipetting errors. It is important to note that Buffer C should be freshly prepared.

| **Reagent** | **µL per mosquito sample** | **96-well plate** |
| --- | --- | --- |
| 1 M Tris-HCl pH 8.00 | 12 | 1272 |
| 0.5 M EDTA pH 8.00 | 3 | 318 |
| 20 mg/mL Proteinase K | 1.2 | 127.2 |
| 5% Tween-20 | 0.6 | 63.6 |
| Molecular grade Water | 43.2 | 4579.2 |
| **Total** | **60** | **6360** |

*Steps:*

1. Add 60 µL of the Buffer C cocktail to each well of the plate.
2. If the mosquitoes are preserved in ethanol, briefly submerge each mosquito in nuclease-free water and then add it to the Buffer C plate using sterile forceps. If the mosquitoes are preserved dry, you can add them directly to the plate.
3. Seal the plate with Flat 8 cap strips (Thermo Scientific, Waltham, MA).
4. Spin down the plate for 1 minute at 1800 rpm.
5. Place damp paper towels inside a sealable plastic bag to increase humidity and limit evaporation. Put the sealed mosquito plate on top of the towels and add two weighted plates on top.
6. Seal the plastic bag and place it in the incubator at 56°C overnight, approximately 16-20 hours*.*

Day 2: Diluting the Lysate and/or Lysate Purification (DNA extraction)

Lysate Dilution:

1. Centrifuge the plate at 1800 rpm for 10 minutes at room temperature.
2. Carefully remove the Flat 8 cap strips (Thermo Scientific, Waltham, MA).
3. Transfer the lysate from the current plate to a new plate. The mosquito carcass plate can be preserved at -20°C.
4. Prepare a 1:10 dilution of lysate (with a final volume 30-40 µL). Pipette to mix thoroughly.
5. Store both the 1:10 dilution of lysate and the remaining lysate at -20°C.

*Lysate purification (DNA Extraction)*

Lysate purification for DNA extraction is carried out using the Thermo Scientific™ KingFisher™ Flex Purification system along with the HighPrep™ Blood & Tissue DNA kit (Magbio Genomics, Gaithersburg, )

**NOTE**: **Ensure that beads are equilibrated to room temperature before use.**

Prepare five KingFisher^TM^ 96-Well Deep Plates as described in Table 2.

**Table 2. KingFisher plate preparation**. Volumes are given per well.

| **Plate Number** | **Volume (µL) per well** |
| --- | --- |
| 1. Sample Plate | 50 µL of lysate + 150 µL MB Solution + 510 µL Binding Bead Mix (200 µL AS Buffer + 10 µL Mag S-1 Beads + 300 µL 100% EtOH) |
| 2. Wash 1 Plate | 400 µL of HSW Buffer |
| 3. Wash 2 Plate | 400 µL of 70% EtOH |
| 4. Wash 3 Plate | 400 µL of 70% EtOH |
| 5. Elution plate | 50 µL of MB Elution Buffer |
| Deep well tip | A Deep well tip comb inserted into a KingFisher standard plate. |

1. Load the KingFisher™ Flex Purification system following the instructions provided below: Collect Count: Set the number of times the magnet goes into the wells. The default value is 3, but it can be adjusted based on your requirements, with a maximum of 5. Collect Time: This is the amount of time the magnetic tips remain at the bottom of the wells. The default value is 1 second. It can range from 1 to 30 seconds. Because the rods are only magnetic at the tips, the magnet will move very slowly through the liquid column in order to ensure all beads are given enough time to be attracted to the tips.
   1. **Binding step:** Release Beads: 00:00:00; Mixing/Heating Parameters: 00:05:00 Fast; Collect Beads count= 5 and Collect time= 0

**Note:** it is recommended to increase Collect Count and/or Collect Time if the sample is “dirty,” e.g., soil, stool or tissue lysates, or for samples that may contain a significant amount of debris or extraneous biochemicals and macromolecules.

- 1. **Collect Beads step:** Collect count = 5 and Collect time = 0
  2. **Wash 1 step:** Release Beads: 00:00:20 (Speed: Bottom Mix). Mixing/Heating Parameters: 00:00:10; bottom mix, 00:00:10; fast, Loop count = 3. Collect beads count = 5 and Collect time = 0.
  3. **Wash 2 step:** Release Beads: 00:00:20; Speed: Bottom Mix. Mixing/Heating Parameters: 00:00:10; bottom mix, 00:00:10; fast, Loop count = 3. Collect beads count = 5 and Collect time = 0.
  4. **Wash 3 step:** Release Beads: 00:00:20; Speed: Fast. Mixing/Heating Parameters: 00:00:10; bottom mix, 00:00:10; fast, Loop count = 2. Collect beads count = 4 and Collect time = 1
  5. **Wash 4 step:** Release Beads: 00:00:00; Speed: Fast. Mixing/Heating Parameters: 00:00:30; fast, Loop count = 1. Collect beads count = 4 and Collect time = 1
  6. **Dry step:** Dry time: 00:02:00 and Tip Position = outside well/tube.
  7. **Elution:** Mixing/Heating Parameters: 00:00:15; bottom mix, 00:00:45; medium, Loop count = 6, Heating during mixing, Preheat.

Block Temperature 75°C, Collect beads count = 5 and Collect time = 30

- 1. **Collect beads:** Mixing/heating parameters: 00:02:00 ; slow
  2. **Leave: Tip Comb Plate**

## Round one PCR (PCR1) Master mix and thermocycler conditions

**Table 3. Optimized round one PCR reagents for each Amplicon Loci**. Primers included the Nextera adapter appended. A total volume was 25 µL per PCR reaction. Abbreviations: **Fwd**: Forward primer; **Rev**: Reverse primer; **MG-H_2_O:** Molecular grade water**; dil-lys:** 1:10 diluted lysate; **NA**: Not Applicable.

| **Amplicon purpose** | **Amplicon Locus** | **Master Mix Reagents (µL)** | | | | | |
| --- | --- | --- | --- | --- | --- | --- | --- |
|  |  | **CloneAmp (µL)** | **Fwd 10nM (µL)** | **Rev1 10nM (µL)** | **Rev2 10nM (µL)** | **MG-H_2_O (µL)** | **Dil-lys (µL)** |
| Mosquito species ID | *ITS2* | 12.5 | 1 | 1 | NA | 8.5 | 2 |
|  | *COX1* | 12.5 | 1 | 1 | NA | 8.5 | 2 |
| Blood Meal ID | *cytB* | 12.5 | 0.7 | 0.7 | NA | 9.1 | 2 |
|  | *16S* | 12.5 | 0.7 | 0.7 | NA | 9.1 | 2 |
| Nectar Meal ID | *rbcL* | 12.5 | 1 | 1 | NA | 8.5 | 2 |
| *Plasmodium* detection | *18S* | 12.5 | 1 | 1 | NA | 8.5 | 2 |
| Insecticide Resistance | *ace-1* | 12.5 | 0.7 | 0.7 | NA | 9.1 | 2 |
|  | *vgsc* | 12.5 | 0.7 | 0.7 | 0.7 | 8.4 | 2 |

**Table 3. Optimized round one PCR Thermocycler conditions for each Amplicon Locus**. Temperatures, times and number of cycles are provided. Abbreviations: **Denat**: Denaturation; **Cyc**: Number of cycles; **Elong.:** Elongation step.

| Locus | Temperature and Time for: | | | | | |  |
| --- | --- | --- | --- | --- | --- | --- | --- |
|  | Initial Denat. | Cyc | Denat. | Annealing | Elong. | Final Elong. | Hold Temp |
| *ITS2* | 95 °C – 01:00 | 34X | 98 °C – 00:10 | 55 °C – 00:15 | 72 °C – 00:45 | 25 °C – 05:00 | 4 °C |
| *COX1* | 98 °C – 03:00 | 34X | 98 °C – 00:10 | 50 °C – 00:45 | 72 °C – 00:45 | 72 °C – 05:00 | 4 °C |
| *CytB* | 95 °C – 01:00 | 34X | 98 °C – 00:10 | 55 °C – 00:15 | 72 °C – 00:15 | 72 °C – 05:00 | 4 °C |
| *16S* | 95 °C – 01:00 | 34X | 98 °C – 00:10 | 55 °C – 00:15 | 72 °C – 00:15 | 72 °C – 05:00 | 4 °C |
| *rbcL* | 95 °C – 03:00 | 35X | 98 °C – 00:20 | 56 °C – 00:30 | 72 °C – 00:15 | 72 °C – 00:30 | 4 °C |
| *18S* | 95 °C – 03:00 | 35X | 98 °C – 00:20 | 57 °C – 00:15 | 72 °C – 00:15 | 72 °C – 01:00 | 4 °C |
| *ace-1* | 95 °C – 03:00 | 10X | 98 °C – 00:10 | 0.5Δ 50-45 °C – 00:45 | 72 °C – 01:00 | 72 °C – 05:00 | 4 °C |
|  |  | 25X | 98 °C – 00:10 | 45 °C – 00:45 | 72 °C – 01:00 |  |  |
| *vgsc* | 95 °C – 03:00 | 10X | 98 °C – 00:10 | 0.5Δ 50-45 °C – 00:45 | 72 °C – 01:00 | 72 °C – 05:00 | 4 °C |
|  |  | 25X | 98 °C – 00:10 | 45 °C – 00:45 | 72 °C – 01:00 |  |  |

## Round two PCR (PCR2) Master mix and thermocycler conditions

For the Illumina dual indexing step (as shown in Fig 1), the PCR2 was conducted using the following approach:

PCR1 products were pooled per sample to perform PCR2, with equal volumes of 5 uL from each PCR1 product. Our final strategy for PCR2 indexing and single MiSeq runs was as follows: *ITS2* and *COX1* were pooled, the *vgsc* gene was indexed and run on the MiSeq individually, and the remaining amplicons (*16s*, *cytB*, *rbcL*, *18S*, *ace-1*) were pooled for PCR2 indexing and run on a single MiSeq. This pooling indexing strategy was developed because the sequencing data yield was biased when short-size amplicons were included in the same DNA library as larger-size amplicons. While the effect of pooling on the efficiency of PCR2 was not specifically tested, the strategy employed yielded good sequencing results, indicating effective amplification and sequencing.

All indexing reactions (PCR2) were carried out in 12 µL containing 5 µL KAPA HiFi HotStart ReadyMix (2X), 2 µL of Unique Dual Index (10 µM) and 5 µL PCR1 product. The indices used in PCR2 were custom-designed by Integrated DNA Technologies (IDT). The optimized PCR2 conditions are: initial denaturation temperature at 95 °C for 1 min, followed by 7 cycles of denaturation at 95 °C for 15 s, annealing at 55 °C for 15 s, and elongation at 72 °C for 30 s; with a final elongation step at 72 °C for 1 min. Three microliters of each PCR2 product (amplicons pooled per sample) were combined into a 1.5 mL microcentrifuge tube for amplicon purification and size selection. Both PCR1 and PCR2 reactions were performed using the VeritiTM 96-Well Thermal Cycler, from Applied Biosystems by Thermo Fisher Scientific.

## Two-tailed bead clean up.

*Pool PCR2 products before beginning

*Allow 30 min for Ampure beads to equilibrate to room temperature

**Table 4**: **Expected fragment size of each amplicon**. Expected size includes adapters and index appended.

| **Amplicon Loci** | **Expected fragment size (bp)** |
| --- | --- |
| *COX1* | 793 |
| *ITS2* | 533-684 |
| *16S* | 233 |
| *18S* | 257-267 |
| *Cytb* | 460 |
| *Ace-1* | 294 |
| *VGSC* | 353- >500 |
| *rbcl* | 472 |

**Protocol:**

1. Perform a 0.9x cleanup: This cleanup removes primer dimer products. The primer-dimers (fragments <200bp) will be at the supernatant that will be discarded. The rest of the DNA will be in the pellet (fragments >200bp).
   1. Combine 100 µL of PCR2 pool with 90 µL beads
   2. Wait 5 min
   3. Place sample on magnet
   4. Once beads have formed the pellet, remove supernatant without disturbing the pellet and discard the supernatant.
   5. Wash twice with ~200 µL of 80% EtOH
   6. Wait 30 sec in each wash. Make sure to not disturb the pellet.
   7. Elute the pellet in 100 µL Tris-HCl (10 mM) and wait 2 minutes.
2. Two-tailed cleanup: This step selects the amplicon fragment of interest (16S in this case, 253bp). Because it is two-tailed cleanup, we will describe the two steps below:
   1. **Step 1**, **0.55x cleanup:**
      1. Combine 100 µL of product from step 1 with 55 µL beads. **Note:** **Fragments >300bp will be at the pellet, whereas fragments <300bp will be at the supernatant**.
      2. Wait 5 minutes
      3. Place sample on magnet
      4. Once beads have formed the pellet, remove supernatant without disturbing the pellet and transfer the supernatant to a new tube.
   2. **Step 2, 0.8x cleanup**:
      1. Combine total volume of product from step 2 with 25 µL beads. The two tailed cleanup is additive, thus the amount of beads added on step 1 adds to step 2. Then 55 + 25 = 80 that corresponds to the volume needed to do a 0.8X cleanup. **Note: Because the supernatant has fragments between 200 and 300bp, the 0.8X cleanup will make sure such fragments in that range are kept in the pellet.**
      2. Wait 5 min
      3. Place sample on magnet
      4. Once beads have formed pellet, remove supernatant and discard
      5. Wash twice the pellet with ~200 µL of 80% EtOH. Make sure to not disturb the pellet.
      6. Wait 30 sec in each wash. Make sure to not disturb the pellet.
      7. Elute in 100 µL Tris-HCl (10 mM) and wait 2 minutes.
3. Perform a 0.8x cleanup (extra clean up to make sure we are keeping the fragments between 200 and 300bp.
   1. Combine 100 µL of product from step 2b with 80 µL beads
   2. Wait 5 min
   3. Place sample on magnet
   4. Once beads have formed pellet, remove supernatant and discard
   5. Wash twice with ~200 µL of 80% EtOH. Make sure to not disturb the pellet.
   6. Wait 30 sec in each wash. Make sure to not disturb the pellet.
   7. Elute in 50 µL Tris-HCl (10 mM) and wait 2 minutes.
   8. Place on magnetic stand until solution clears
   9. Collect supernatant.
4. Perform a 0.7x cleanup
   1. Combine 42 µL beads with 60 µL well-mixed PCR2 product. Incubate at room temperature for 5 minutes.
   2. Place on a magnetic stand for 3 minutes. Discard the supernatant without disturbing the bead pellet.
   3. While still on the magnetic stand, add 180 µL of fresh 80% ethanol to the beads and incubate for 30 seconds.  Remove supernatant and discard.  Repeat this wash once.
   4. While still on the magnetic stand, remove any residual ethanol with a small (e.g., p20) pipette and/or allow ethanol to evaporate for 2 minutes (with tubes uncovered). Do not exceed 2 minutes of evaporation time.
   5. Remove from the magnetic stand and add 30 µL 10 mM Tris-Cl, pH 8.5. Mix thoroughly and let incubate at room temperature for 5 minutes.
   6. Place on the magnetic stand for 3 minutes.
   7. Collect 30 µL supernatant without disturbing the pellet.
   8. Add 21 µL beads to 30 µL supernatant from step g and mix for repeat 0.7x bead cleanup. Incubate for 5 minutes.
   9. Repeat steps b through d.
   10. Remove from the magnetic stand and add 15.5 µL 10 mM Tris-Cl, pH 8.5. Mix thoroughly and let incubate at room temperature for 5 minutes.
   11. Place on the magnetic stand for 3 minutes.
   12. Collect 13 µL supernatant without disturbing the pellet.

# **S2 Text: Empirical data informing filtering criteria**


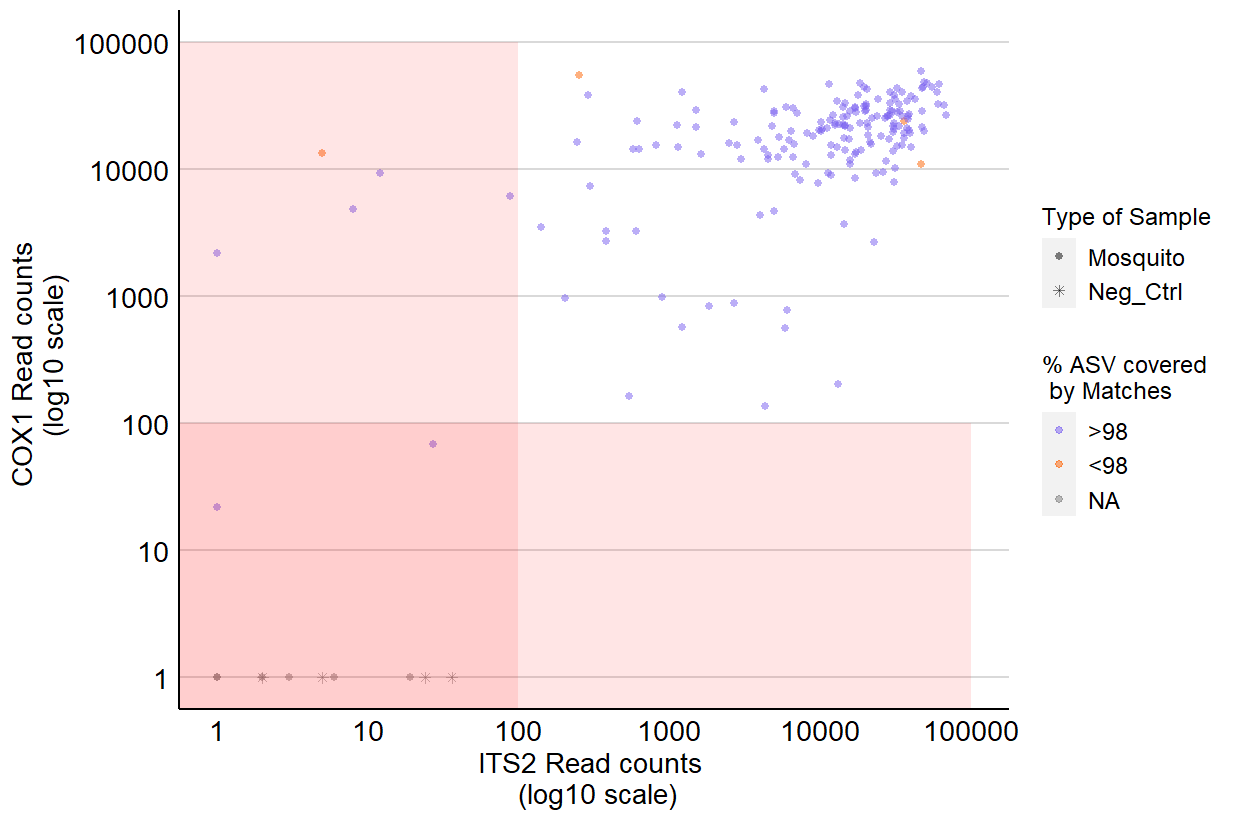


**Figure 1. Read Count Support for Batch 1.** Different shapes highlight the type of sample analyzed and the point colors code highlights the BLAST % support. The area shaded represents a potential threshold to exclude potentially contaminated samples. The total number of samples included in this batch was 188, in addition to four negative controls.

**Batch1**

Negative controls in Batch 1 were Buffer C from the lysis process (2 replicates) and water for PCR1 (2 replicates). The buffer C controls showed 23 and 35 read counts. Water controls showed 1-5 read counts.

**Batch 4**

Negative controls in Batch 1 were Buffer C from the lysis process (2 replicates) and water for PCR1 step (2 replicates). The buffer C controls showed 0 and 1 read counts. Water controls showed 7 and 10 read counts.

# **S3 Text: Supplemental bioinformatic methods**

## Database Curation

Annotated sequences were pulled down from the BOLD database and from NCBI NT with relevant Genbank text annotations [1]. For sequences from NCBI, specific keywords such as [COX1; mitochondrial; diptera] were utilized to retrieve relevant sequences using Entrez tools. These annotated references were searched against the NT database on January 26th, 2023 using BLASTN v2.9.0. The search was taxon-limited to order diptera (*COX1*, *ITS2*), clade tetrapoda (*16S*, *cytb*), and clade tracheophyta (*rbcL*). Due to the presence of nuclear paralogs for *16S* and *cytb*, mitochondrial references were filtered to ensure they came from a nominal “mitochondrial” source using NCBI annotations. Each unique hit to a database reference was extracted along with 100bp flanking regions.

The untrimmed references were searched for both of the PCR1 primer sequences, allowing for up to 3 mismatches in order to extract only the region that would be amplified and exclude any references not homologous to the amplified region. Any references with ambiguous bases such as Ns were filtered out.

References were kept that fell within the expected fragment length distribution for known size-conserved loci amplicon. References belonging to extinct taxa annotated by the NCBI Taxonomy database were filtered out [2, 3].

A reciprocal BLAST was performed for each reference database to find mislabels [4]. Identical reference sequences matching to divergent taxa above the genus rank were removed.

Multiple sequence alignments for *COX1*, *cytb*, and *rbcL* were generated using MAFFT “fftns --op 10 --leavegappyregion”. Alignments were inspected for gaps caused by single references and those references were removed [7].

Maximum likelihood phylogenetic trees were estimated using FastTree v2.1.8 with model GTRCAT [5]. The reference phylogeny was checked for long branches and trimmed if the branch length was greater than 1. Due to the presence of mislabels in publicly available databases, genus-level phylogenetic relationships were assessed. If a genus was polyphyletic or found in different regions of the phylogeny due to a clearly identifiable mislabel, then the problematic reference was removed.

## Taxonomy Assignment

For a given Amplicon Sequence Variant (ASV), our VecTreeID pipeline reports both the closest match(es) in the database using BLAST (BLAST Top Hit) and the phylogenetic clade containing all plausible matches (EPA Confidence Clade). (Fig 7, main text). Not all ASVs will receive assignments via both methods.

VecTreeID processes paired-end Illumina sequencing data in the form of FASTQ files using a custom analysis pipeline that utilizes the Divisive Amplicon Denoising Algorithm (DADA2) tool designed to obtain microhaplotypes as previously described in AMPLseq amplicon panel for parasite surveillance [6]. VecTreeID requires the name of the amplicon, and a dada2 directory containing at least the seqtab and bimera files.

Furthermore, BLAST-Tree takes in various filtration parameters to ensure the quality of the assignment. The seqtab table is filtered based on predicted bimera status, expected length, and read counts. ASVs that are too small or too large are removed. ASVs that are below --min_asv_readcount are not given taxonomy assignments and deemed artefactual. Samples that are below --min_sample_readcount are not given sample-level assignments. The empirical read count thresholds are based on the validation experiments previously described for the limitation of detection amongst the bionomic amplicons.

One aspect of ASVs that needs to be addressed is the merging and concatenation of ASVs. The read length of sequencing technology can limit the total amount of bases sequenced during PCR2 from the PCR1 amplicon region. Depending on the length conservation of the amplicon and sequencing technology read length, the ASVs will have overlapping or separate forward and reverse reads. For example, MiSeq Illumina sequencers have 250bp reads, while iSeq Illumina sequencers have 150bp reads. This will lead to amplicon sequence variants which are either merged or concatenated. Merged ASVs have their first and second reads combined at the overlap, while concatenated ASVs have the internal region padded with Ns.

For an amplicon that is greater than 500bps, the ASVs would be merged with the MiSeq length reads while the iSeq length reads would be concatenated. Depending on the sequencer used, this can impact the total number of informative sites in an amplicon sequence variant, which in turn can increase the ambiguity in taxonomic assignment.

ASVs are assessed using BLASTN v2.9.0 ( -max_hsps 10, -max_target_seqs 10) against the reference databases and non-contiguous alignments are merged. If the top BLASTN match to an ASV is below the percent identity --artefact_cutoff (default: 0.80 ), then it is identified as an artifact and removed from subsequent assignments. If this match is below the percent identity --min_coverage (default: 0.95 ) and --min_identity (default: 0.97 ), then no species-level assignment is given. All ASVs that pass the artifact filter are promoted to phylogenetic-based assignment, including those that failed to get a species-level assignment.

MAFFT v7.520 (--addfragments, --6merpair) is used to generate a multiple sequence alignment of ASVs to the reference databases. EPA-ng v0.3.8 (--filter-acc-lwr 0.99 , --filter-max 50 , --model GTR+G) is used for phylogenetic placements of short reads with the reference multiple sequence alignment and ASV multiple sequence alignment as inputs [8]. The descendant leaves of all EPA-ng hits are defined as possible taxon assignments.

The pipeline determines the convergent Linnaean rank of hits to best capture the ambiguity in taxonomic assignment by leveraging the NCBI Taxonomy database. Every single reference has a taxid associated with it, and a corresponding taxonomic lineage as previously defined by a taxonomist. In the case of more than one potential taxonomic assignment, then the Linnaean rank (ie; species or genus) that contains all assignments is reported back. This enables a format for reporting both an assignment in the form of the taxon and ambiguity in the form of rank.

In order to generate an interpretable assignment given the possibility of multiple species labels, the Linnean Convergent Rank for all EPA-ng hits is determined. Annotated Linnean taxonomic ranks for each reference are retrieved from the NCBI taxonomy database, and the rank label which contains all hits is phylogenetic assignment.

**References Cited**

1. Ratnasingham, S., & Hebert, P. D. (2007). BOLD: The Barcode of Life Data System (<http://www>. barcodinglife. org). Molecular ecology notes, 7(3), 355-364.
2. Schoch, Conrad L., et al. "NCBI Taxonomy: a comprehensive update on curation, resources and tools." Database 2020 (2020): baaa062.
3. <https://www.ncbi.nlm.nih.gov/Taxonomy/taxonomyhome.html/index.cgi?chapter=extinct>
4. Shah, Nidhi, Stephen F. Altschul, and Mihai Pop. "Outlier detection in BLAST hits." Algorithms for Molecular Biology 13.1 (2018): 1-9.
5. Price, Morgan N., Paramvir S. Dehal, and Adam P. Arkin. "FastTree 2–approximately maximum-likelihood trees for large alignments." PloS one 5.3 (2010): e9490.
6. LaVerriere, E., Schwabl, P., Carrasquilla, M., Taylor, A. R., Johnson, Z. M., Shieh, M., ... & Neafsey, D. E. (2022). Design and implementation of multiplexed amplicon sequencing panels to serve genomic epidemiology of infectious disease: a malaria case study. Molecular ecology resources, 22(6), 2285-2303.
7. Katoh, Kazutaka, and Daron M. Standley. "MAFFT multiple sequence alignment software version 7: improvements in performance and usability." Molecular biology and evolution 30.4 (2013): 772-780.
8. Barbera, Pierre, et al. "EPA-ng: massively parallel evolutionary placement of genetic sequences." Systematic biology 68.2 (2019): 365-369.

# **S4 Text: Assessment of taxonomic breadth**

To assess the taxonomic breadth, the successful amplification of representative taxa within their designated taxonomic detection range was employed. For the agarose gel (1.2%), PCR1 products were loaded. These products were amplified from male mosquitoes samples (*Anopheles albimanus*, *An. stephensi,* *Culex pipiens* and *Aedes aegypti*) using a 1:10 lysate dilution as the DNA template. Each well was loaded with a total volume of ten microliters, comprising three microliters of PCR1 product mixed with seven microliters of loading buffer.

In Figure 1, the taxonomic coverage of *ITS2*, *cytB* and *COX1* is demonstrated through agarose gel electrophoresis. *COX1* and *ITS2* exhibited successful PCR product amplification across all mosquito species tested. However, *cytB* was only successfully amplified in *Anopheles* species.


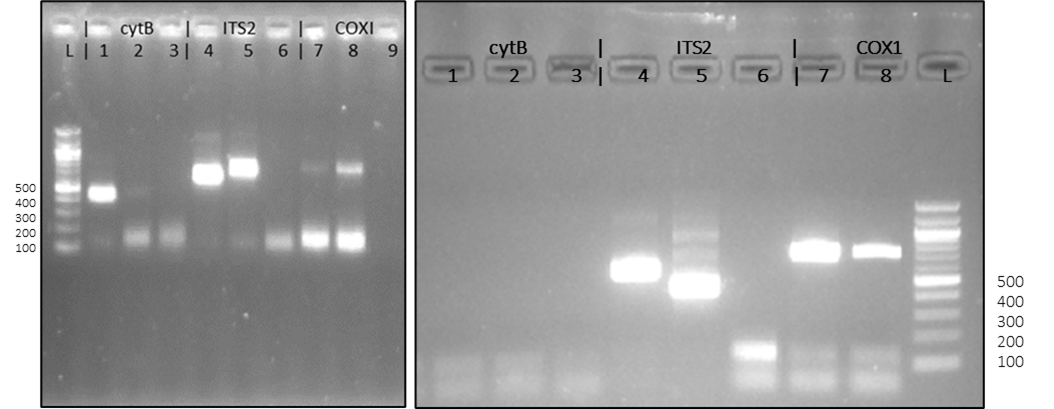


**Figure 1**. **Electrophoresis gels illustrating Taxonomic breadth**. *Left gel*: In this gel, Lane 1 functions as the positive control and includes a 100bp DNA ladder (L) with known fragment sizes. Lane labeled as “1” is *Anopheles albimanus*, Lane “2” *Anopheles stephensi* and Lane “3” represents the Negative PCR control (water). *Right gel:* The last lane of this gel serves as the positive control and includes a 100bp DNA ladder (L). Lane “1” represents the Negative PCR control (water), Lane “2” *Culex pipiens* and Lane “3” *Aedes aegypti.*

In Figure 2, the taxonomic coverage of *rbcL* is demonstrated through agarose gel electrophoresis. *rbcL* gene exhibited successful PCR product amplification across all plant species tested. The taxonomic coverage for the remaining loci is depicted in Figure 3 of the main manuscript text.

*
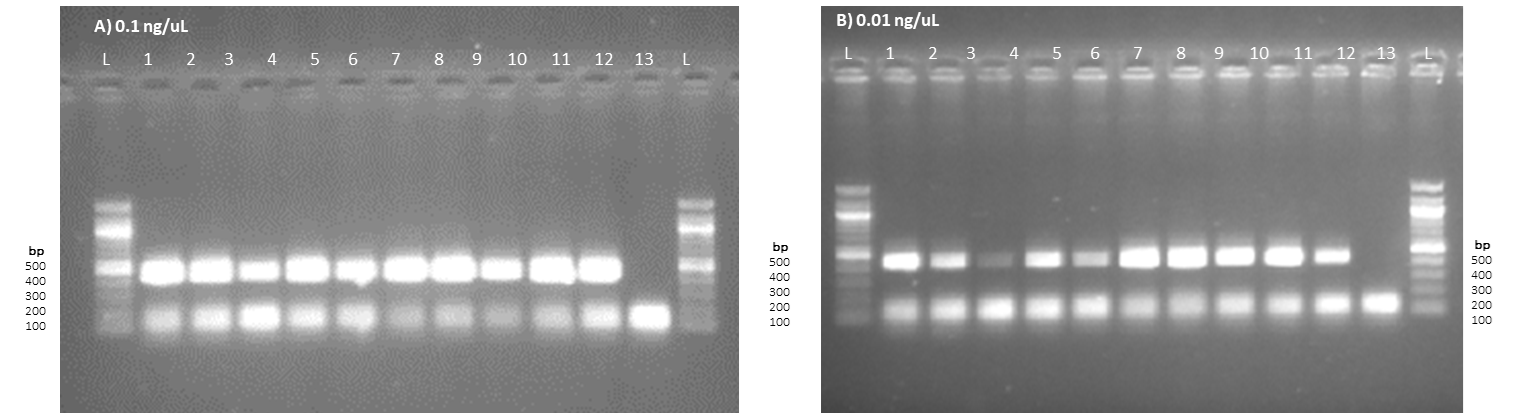
***Figure 2**. **Electrophoresis gels illustrating taxonomic breadth for *rbcL* gene**. *Left gel*: This gel displays PCR products utilizing 0.1 ng/uL as DNA templates. In this gel, Lane 1 and 15 functions as the positive control and includes a 100bp DNA ladder (L) with known fragment sizes. The samples are represented as displayed as follows: 1) *Mimosa pudica*, 2) *Epiphyllum phyllantus*, 3) *Cattleya percivaliana*, 4) *Aechmea melinonii*, 5) *Monstera deliciosa*, 6) *Persea americana*, 7) *Aristolochia gigantea*, 8) *Passiflora edulis*, 9) *Heliconia rostrata*, 10) *Psychotria viridis*, 11) *Mimosa pudica-2*, 12) *Epiphyllum phyllantus-2* and 13) Negative PCR control (water). *Right gel:* PCR products using 0.01 ng/uL as DNA template.
